# Supplementary material for: Allelopathic interactions of Carthamus oxyacantha, Macrophomina phaseolina and maize: Implications for the use of Carthamus oxyacantha as a natural disease management strategy in maize
Source: PLoS One. 2024 Oct 31;19(10):e0307082. doi: 10.1371/journal.pone.0307082 (PMC11527155; doi:10.1371/journal.pone.0307082)
Supplement: S1 File — (DOCX) [file pone.0307082.s001.docx]

**S1. ANOVA file for the effect of treatments on the disease incidence (DI) and area under disease progress curve disease incidence (AUDPC DI) on maize plants.**

42 DAYS

**One-way ANOVA: 42 days versus treatments**

**Method**

| Null hypothesis | All means are equal |
| --- | --- |
| Alternative hypothesis | Not all means are equal |
| Significance level | α = 0.05 |

*Equal variances were assumed for the analysis.*

**Factor Information**

| **Factor** | **Levels** | **Values** |
| --- | --- | --- |
| treatments | 19 | AMp1, AMp2, AMp3, C, Co1, Co2, Co3, Mp1, Mp1+Co1, Mp1+Co2, Mp1+Co3, Mp2, Mp2+Co1, Mp2+Co2, Mp2+Co3, Mp3, Mp3+Co1, Mp3+Co2, Mp3+Co3 |

**Analysis of Variance**

| **Source** | **DF** | **Seq SS** | **Contribution** | **Adj SS** | **Adj MS** | **F-Value** | **P-Value** |
| --- | --- | --- | --- | --- | --- | --- | --- |
| treatments | 18 | 96842 | 46.37% | 96842 | 5380 | 3.65 | 0.000 |
| Error | 76 | 112000 | 53.63% | 112000 | 1474 |  |  |
| Total | 94 | 208842 | 100.00% |  |  |  |  |

**Model Summary**

| **S** | **R-sq** | **R-sq(adj)** | **PRESS** | **R-sq(pred)** |
| --- | --- | --- | --- | --- |
| 38.3886 | 46.37% | 33.67% | 175000 | 16.20% |

56 DAYS

**One-way ANOVA: 56 days versus treatments**

**Method**

| Null hypothesis | All means are equal |
| --- | --- |
| Alternative hypothesis | Not all means are equal |
| Significance level | α = 0.05 |

*Equal variances were assumed for the analysis.*

**Factor Information**

| **Factor** | **Levels** | **Values** |
| --- | --- | --- |
| treatments | 19 | AMp1, AMp2, AMp3, C, Co1, Co2, Co3, Mp1, Mp1+Co1, Mp1+Co2, Mp1+Co3, Mp2, Mp2+Co1, Mp2+Co2, Mp2+Co3, Mp3, Mp3+Co1, Mp3+Co2, Mp3+Co3 |

**Analysis of Variance**

| **Source** | **DF** | **Seq SS** | **Contribution** | **Adj SS** | **Adj MS** | **F-Value** | **P-Value** |
| --- | --- | --- | --- | --- | --- | --- | --- |
| treatments | 18 | 99368 | 46.14% | 99368 | 5520 | 3.62 | 0.000 |
| Error | 76 | 116000 | 53.86% | 116000 | 1526 |  |  |
| Total | 94 | 215368 | 100.00% |  |  |  |  |

**Model Summary**

| **S** | **R-sq** | **R-sq(adj)** | **PRESS** | **R-sq(pred)** |
| --- | --- | --- | --- | --- |
| 39.0681 | 46.14% | 33.38% | 181250 | 15.84% |

70 DAYS

**One-way ANOVA: 70 days versus Treatments**

**Method**

| Null hypothesis | All means are equal |
| --- | --- |
| Alternative hypothesis | Not all means are equal |
| Significance level | α = 0.05 |

*Equal variances were assumed for the analysis.*

**Factor Information**

| **Factor** | **Levels** | **Values** |
| --- | --- | --- |
| Treatments | 19 | AMp1, AMp2, AMp3, C, Co1, Co2, Co3, Mp1, Mp1+Co1, Mp1+Co2, Mp1+Co3, Mp2, Mp2+Co1, Mp2+Co2, Mp2+Co3, Mp3, Mp3+Co1, Mp3+Co2, Mp3+Co3 |

**Analysis of Variance**

| **Source** | **DF** | **Seq SS** | **Contribution** | **Adj SS** | **Adj MS** | **F-Value** | **P-Value** |
| --- | --- | --- | --- | --- | --- | --- | --- |
| Treatments | 18 | 121895 | 53.02% | 121895 | 6772 | 4.77 | 0.000 |
| Error | 76 | 108000 | 46.98% | 108000 | 1421 |  |  |
| Total | 94 | 229895 | 100.00% |  |  |  |  |

**Model Summary**

| **S** | **R-sq** | **R-sq(adj)** | **PRESS** | **R-sq(pred)** |
| --- | --- | --- | --- | --- |
| 37.6969 | 53.02% | 41.90% | 168750 | 26.60% |

AUDPC DI

**One-way ANOVA: AUDPC DI versus Treatments**

**Method**

| Null hypothesis | All means are equal |
| --- | --- |
| Alternative hypothesis | Not all means are equal |
| Significance level | α = 0.05 |

*Equal variances were assumed for the analysis.*

**Factor Information**

| **Factor** | **Levels** | **Values** |
| --- | --- | --- |
| Treatments | 19 | AMp1, AMp2, AMp3, C, Co1, Co2, Co3, Mp1, Mp1+Co1, Mp1+Co2, Mp1+Co3, Mp2, Mp2+Co1, Mp2+Co2, Mp2+Co3, Mp3, Mp3+Co1, Mp3+Co2, Mp3+Co3 |

**Analysis of Variance**

| **Source** | **DF** | **Seq SS** | **Contribution** | **Adj SS** | **Adj MS** | **F-Value** | **P-Value** |
| --- | --- | --- | --- | --- | --- | --- | --- |
| Treatments | 18 | 80215579 | 50.95% | 80215579 | 4456421 | 4.39 | 0.000 |
| Error | 76 | 77224000 | 49.05% | 77224000 | 1016105 |  |  |
| Total | 94 | 157439579 | 100.00% |  |  |  |  |

**Model Summary**

| **S** | **R-sq** | **R-sq(adj)** | **PRESS** | **R-sq(pred)** |
| --- | --- | --- | --- | --- |
| 1008.02 | 50.95% | 39.33% | 120662500 | 23.36% |
