# Supplementary material for: Allelopathic interactions of Carthamus oxyacantha, Macrophomina phaseolina and maize: Implications for the use of Carthamus oxyacantha as a natural disease management strategy in maize
Source: PLoS One. 2024 Oct 31;19(10):e0307082. doi: 10.1371/journal.pone.0307082 (PMC11527155; doi:10.1371/journal.pone.0307082)
Supplement: S2 File — (DOCX) [file pone.0307082.s002.docx]

**S2. ANOVA file for the effect of treatments on the disease severity (DS) and area under disease progress curve disease severity (AUDPC DS) on maize plants.**

42 DAYS

**One-way ANOVA: 42 days versus Treatments**

**Method**

| Null hypothesis | All means are equal |
| --- | --- |
| Alternative hypothesis | Not all means are equal |
| Significance level | α = 0.05 |

*Equal variances were assumed for the analysis.*

**Factor Information**

| **Factor** | **Levels** | **Values** |
| --- | --- | --- |
| Treatments | 19 | AMp1, AMp2, AMp3, C, Co1, Co2, Co3, Mp1, Mp1+Co1, Mp1+Co2, Mp1+Co3, Mp2, Mp2+Co1, Mp2+Co2, Mp2+Co3, Mp3, Mp3+Co1, Mp3+Co2, Mp3+Co3 |

**Analysis of Variance**

| **Source** | **DF** | **Seq SS** | **Contribution** | **Adj SS** | **Adj MS** | **F-Value** | **P-Value** |
| --- | --- | --- | --- | --- | --- | --- | --- |
| Treatments | 18 | 17255 | 52.90% | 17255 | 958.6 | 4.74 | 0.000 |
| Error | 76 | 15360 | 47.10% | 15360 | 202.1 |  |  |
| Total | 94 | 32615 | 100.00% |  |  |  |  |

**Model Summary**

| **S** | **R-sq** | **R-sq(adj)** | **PRESS** | **R-sq(pred)** |
| --- | --- | --- | --- | --- |
| 14.2164 | 52.90% | 41.75% | 24000 | 26.41% |

56 DAYS

**One-way ANOVA: 56 days versus Treatments**

**Method**

| Null hypothesis | All means are equal |
| --- | --- |
| Alternative hypothesis | Not all means are equal |
| Significance level | α = 0.05 |

*Equal variances were assumed for the analysis.*

**Factor Information**

| **Factor** | **Levels** | **Values** |
| --- | --- | --- |
| Treatments | 19 | AMp1, AMp2, AMp3, C, Co1, Co2, Co3, Mp1, Mp1+Co1, Mp1+Co2, Mp1+Co3, Mp2, Mp2+Co1, Mp2+Co2, Mp2+Co3, Mp3, Mp3+Co1, Mp3+Co2, Mp3+Co3 |

**Analysis of Variance**

| **Source** | **DF** | **Seq SS** | **Contribution** | **Adj SS** | **Adj MS** | **F-Value** | **P-Value** |
| --- | --- | --- | --- | --- | --- | --- | --- |
| Treatments | 18 | 34535 | 46.74% | 34535 | 1918.6 | 3.70 | 0.000 |
| Error | 76 | 39360 | 53.26% | 39360 | 517.9 |  |  |
| Total | 94 | 73895 | 100.00% |  |  |  |  |

**Model Summary**

| **S** | **R-sq** | **R-sq(adj)** | **PRESS** | **R-sq(pred)** |
| --- | --- | --- | --- | --- |
| 22.7573 | 46.74% | 34.12% | 61500 | 16.77% |

70 DAYS

**One-way ANOVA: 70 days versus Treatments**

**Method**

| Null hypothesis | All means are equal |
| --- | --- |
| Alternative hypothesis | Not all means are equal |
| Significance level | α = 0.05 |

*Equal variances were assumed for the analysis.*

**Factor Information**

| **Factor** | **Levels** | **Values** |
| --- | --- | --- |
| Treatments | 19 | AMp1, AMp2, AMp3, C, Co1, Co2, Co3, Mp1, Mp1+Co1, Mp1+Co2, Mp1+Co3, Mp2, Mp2+Co1, Mp2+Co2, Mp2+Co3, Mp3, Mp3+Co1, Mp3+Co2, Mp3+Co3 |

**Analysis of Variance**

| **Source** | **DF** | **Seq SS** | **Contribution** | **Adj SS** | **Adj MS** | **F-Value** | **P-Value** |
| --- | --- | --- | --- | --- | --- | --- | --- |
| Treatments | 18 | 72733 | 57.28% | 72733 | 4040.7 | 5.66 | 0.000 |
| Error | 76 | 54240 | 42.72% | 54240 | 713.7 |  |  |
| Total | 94 | 126973 | 100.00% |  |  |  |  |

**Model Summary**

| **S** | **R-sq** | **R-sq(adj)** | **PRESS** | **R-sq(pred)** |
| --- | --- | --- | --- | --- |
| 26.7149 | 57.28% | 47.16% | 84750 | 33.25% |

AUDPC

**One-way ANOVA: AUDPS(DS) versus Treatments**

**Method**

| Null hypothesis | All means are equal |
| --- | --- |
| Alternative hypothesis | Not all means are equal |
| Significance level | α = 0.05 |

*Equal variances were assumed for the analysis.*

**Factor Information**

| **Factor** | **Levels** | **Values** |
| --- | --- | --- |
| Treatments | 19 | AMp1, AMp2, AMp3, C, Co1, Co2, Co3, Mp1, Mp1+Co1, Mp1+Co2, Mp1+Co3, Mp2, Mp2+Co1, Mp2+Co2, Mp2+Co3, Mp3, Mp3+Co1, Mp3+Co2, Mp3+Co3 |

**Analysis of Variance**

| **Source** | **DF** | **Seq SS** | **Contribution** | **Adj SS** | **Adj MS** | **F-Value** | **P-Value** |
| --- | --- | --- | --- | --- | --- | --- | --- |
| Treatments | 18 | 28579276 | 54.25% | 28579276 | 1587738 | 5.01 | 0.000 |
| Error | 76 | 24100160 | 45.75% | 24100160 | 317107 |  |  |
| Total | 94 | 52679436 | 100.00% |  |  |  |  |

**Model Summary**

| **S** | **R-sq** | **R-sq(adj)** | **PRESS** | **R-sq(pred)** |
| --- | --- | --- | --- | --- |
| 563.123 | 54.25% | 43.42% | 37656500 | 28.52% |
