# Supplementary material for: Allelopathic interactions of Carthamus oxyacantha, Macrophomina phaseolina and maize: Implications for the use of Carthamus oxyacantha as a natural disease management strategy in maize
Source: PLoS One. 2024 Oct 31;19(10):e0307082. doi: 10.1371/journal.pone.0307082 (PMC11527155; doi:10.1371/journal.pone.0307082)
Supplement: S3 File — (DOCX) [file pone.0307082.s003.docx]

**S3. ANOVA file for the effect of treatments on the morphological attributes of maize.**

SL(A)

**One-way ANOVA: Shoot Length versus Treatments**

**Method**

| Null hypothesis | All means are equal |
| --- | --- |
| Alternative hypothesis | Not all means are equal |
| Significance level | α = 0.05 |

*Equal variances were assumed for the analysis.*

**Factor Information**

| **Factor** | **Levels** | **Values** |
| --- | --- | --- |
| Treatments | 19 | AMp1, AMp2, AMp3, C, Co1, Co2, Co3, Mp1, Mp1+Co1, Mp1+Co2, Mp1+Co3, Mp2, Mp2+Co1, Mp2+Co2, Mp2+Co3, Mp3, Mp3+Co1, Mp3+Co2, Mp3+Co3 |

**Analysis of Variance**

| **Source** | **DF** | **Adj SS** | **Adj MS** | **F-Value** | **P-Value** |
| --- | --- | --- | --- | --- | --- |
| Treatments | 18 | 16230.5 | 901.693 | 92.26 | 0.000 |
| Error | 75 | 733.0 | 9.773 |  |  |
| Total | 93 | 16963.5 |  |  |  |

**Model Summary**

| **S** | **R-sq** | **R-sq(adj)** | **R-sq(pred)** |
| --- | --- | --- | --- |
| 3.12625 | 95.68% | 94.64% | 93.23% |

SDM(B)

**One-way ANOVA: SDM versus Treatments**

**Method**

| Null hypothesis | All means are equal |
| --- | --- |
| Alternative hypothesis | Not all means are equal |
| Significance level | α = 0.05 |

*Equal variances were assumed for the analysis.*

**Factor Information**

| **Factor** | **Levels** | **Values** |
| --- | --- | --- |
| Treatments | 19 | AMp1, AMp2, AMp3, C, Co1, Co2, Co3, Mp1, Mp1+Co1, Mp1+Co2, Mp1+Co3, Mp2, Mp2+Co1, Mp2+Co2, Mp2+Co3, Mp3, Mp3+Co1, Mp3+Co2, Mp3+Co3 |

**Analysis of Variance**

| **Source** | **DF** | **Adj SS** | **Adj MS** | **F-Value** | **P-Value** |
| --- | --- | --- | --- | --- | --- |
| Treatments | 18 | 726.43 | 40.3573 | 94.43 | 0.000 |
| Error | 76 | 32.48 | 0.4274 |  |  |
| Total | 94 | 758.91 |  |  |  |

**Model Summary**

| **S** | **R-sq** | **R-sq(adj)** | **R-sq(pred)** |
| --- | --- | --- | --- |
| 0.653734 | 95.72% | 94.71% | 93.31% |

RDM(C)

**One-way ANOVA: RDM versus Treatments**

**Method**

| Null hypothesis | All means are equal |
| --- | --- |
| Alternative hypothesis | Not all means are equal |
| Significance level | α = 0.05 |

*Equal variances were assumed for the analysis.*

**Factor Information**

| **Factor** | **Levels** | **Values** |
| --- | --- | --- |
| Treatments | 19 | AMp1, AMp2, AMp3, C, Co1, Co2, Co3, Mp1, Mp1+Co1, Mp1+Co2, Mp1+Co3, Mp2, Mp2+Co1, Mp2+Co2, Mp2+Co3, Mp3, Mp3+Co1, Mp3+Co2, Mp3+Co3 |

**Analysis of Variance**

| **Source** | **DF** | **Adj SS** | **Adj MS** | **F-Value** | **P-Value** |
| --- | --- | --- | --- | --- | --- |
| Treatments | 18 | 24.789 | 1.37719 | 70.11 | 0.000 |
| Error | 76 | 1.493 | 0.01964 |  |  |
| Total | 94 | 26.282 |  |  |  |

**Model Summary**

| **S** | **R-sq** | **R-sq(adj)** | **R-sq(pred)** |
| --- | --- | --- | --- |
| 0.140150 | 94.32% | 92.97% | 91.13% |
