# Supplementary material for: Allelopathic interactions of Carthamus oxyacantha, Macrophomina phaseolina and maize: Implications for the use of Carthamus oxyacantha as a natural disease management strategy in maize
Source: PLoS One. 2024 Oct 31;19(10):e0307082. doi: 10.1371/journal.pone.0307082 (PMC11527155; doi:10.1371/journal.pone.0307082)
Supplement: S4 File — (DOCX) [file pone.0307082.s004.docx]

**S4. ANOVA file for the effect of treatments on the pigments of maize.**

CHL A

**One-way ANOVA: Chl a versus Treatments**

**Method**

| Null hypothesis | All means are equal |
| --- | --- |
| Alternative hypothesis | Not all means are equal |
| Significance level | α = 0.05 |

*Equal variances were assumed for the analysis.*

**Factor Information**

| **Factor** | **Levels** | **Values** |
| --- | --- | --- |
| Treatments | 19 | AMp1, AMp2, AMp3, C, Co1, Co2, Co3, Mp1, Mp1+Co1, Mp1+Co2, Mp1+Co3, Mp2, Mp2+Co1, Mp2+Co2, Mp2+Co3, Mp3, Mp3+Co1, Mp3+Co2, Mp3+Co3 |

**Analysis of Variance**

| **Source** | **DF** | **Adj SS** | **Adj MS** | **F-Value** | **P-Value** |
| --- | --- | --- | --- | --- | --- |
| Treatments | 18 | 339.84 | 18.8800 | 51.63 | 0.000 |
| Error | 76 | 27.79 | 0.3657 |  |  |
| Total | 94 | 367.63 |  |  |  |

**Model Summary**

| **S** | **R-sq** | **R-sq(adj)** | **R-sq(pred)** |
| --- | --- | --- | --- |
| 0.604727 | 92.44% | 90.65% | 88.19% |

CHL B

**One-way ANOVA: Chl b versus Treatments**

**Method**

| Null hypothesis | All means are equal |
| --- | --- |
| Alternative hypothesis | Not all means are equal |
| Significance level | α = 0.05 |

*Equal variances were assumed for the analysis.*

**Factor Information**

| **Factor** | **Levels** | **Values** |
| --- | --- | --- |
| Treatments | 19 | AMp1, AMp2, AMp3, C, Co1, Co2, Co3, Mp1, Mp1+Co1, Mp1+Co2, Mp1+Co3, Mp2, Mp2+Co1, Mp2+Co2, Mp2+Co3, Mp3, Mp3+Co1, Mp3+Co2, Mp3+Co3 |

**Analysis of Variance**

| **Source** | **DF** | **Adj SS** | **Adj MS** | **F-Value** | **P-Value** |
| --- | --- | --- | --- | --- | --- |
| Treatments | 18 | 127.10 | 7.0611 | 21.50 | 0.000 |
| Error | 76 | 24.96 | 0.3284 |  |  |
| Total | 94 | 152.06 |  |  |  |

**Model Summary**

| **S** | **R-sq** | **R-sq(adj)** | **R-sq(pred)** |
| --- | --- | --- | --- |
| 0.573036 | 83.59% | 79.70% | 74.36% |

CAROTENOIDS

**One-way ANOVA: Carotenoids versus Treatments**

**Method**

| Null hypothesis | All means are equal |
| --- | --- |
| Alternative hypothesis | Not all means are equal |
| Significance level | α = 0.05 |

*Equal variances were assumed for the analysis.*

**Factor Information**

| **Factor** | **Levels** | **Values** |
| --- | --- | --- |
| Treatments | 19 | AMp1, AMp2, AMp3, C, Co1, Co2, Co3, Mp1, Mp1+Co1, Mp1+Co2, Mp1+Co3, Mp2, Mp2+Co1, Mp2+Co2, Mp2+Co3, Mp3, Mp3+Co1, Mp3+Co2, Mp3+Co3 |

**Analysis of Variance**

| **Source** | **DF** | **Adj SS** | **Adj MS** | **F-Value** | **P-Value** |
| --- | --- | --- | --- | --- | --- |
| Treatments | 18 | 47828 | 2657.11 | 61.83 | 0.000 |
| Error | 76 | 3266 | 42.98 |  |  |
| Total | 94 | 51094 |  |  |  |

**Model Summary**

| **S** | **R-sq** | **R-sq(adj)** | **R-sq(pred)** |
| --- | --- | --- | --- |
| 6.55568 | 93.61% | 92.09% | 90.01% |
