# Supplementary material for: Allelopathic interactions of Carthamus oxyacantha, Macrophomina phaseolina and maize: Implications for the use of Carthamus oxyacantha as a natural disease management strategy in maize
Source: PLoS One. 2024 Oct 31;19(10):e0307082. doi: 10.1371/journal.pone.0307082 (PMC11527155; doi:10.1371/journal.pone.0307082)
Supplement: S5 File — (DOCX) [file pone.0307082.s005.docx]

**S5. ANOVA file for the effect of treatments on (A) rate of carbon assimilation and (B) stomatal conductance of maize.**

A

**One-way ANOVA: A versus Treatments**

**Method**

| Null hypothesis | All means are equal |
| --- | --- |
| Alternative hypothesis | Not all means are equal |
| Significance level | α = 0.05 |

*Equal variances were assumed for the analysis.*

**Factor Information**

| **Factor** | **Levels** | **Values** |
| --- | --- | --- |
| Treatments | 19 | AMp1, AMp2, AMp3, C, Co1, Co2, Co3, Mp1, Mp1+Co1, Mp1+Co2, Mp1+Co3, Mp2, Mp2+Co1, Mp2+Co2, Mp2+Co3, Mp3, Mp3+Co1, Mp3+Co2, Mp3+Co3 |

**Analysis of Variance**

| **Source** | **DF** | **Adj SS** | **Adj MS** | **F-Value** | **P-Value** |
| --- | --- | --- | --- | --- | --- |
| Treatments | 18 | 1829.9 | 101.661 | 49.61 | 0.000 |
| Error | 76 | 155.7 | 2.049 |  |  |
| Total | 94 | 1985.6 |  |  |  |

**Model Summary**

| **S** | **R-sq** | **R-sq(adj)** | **R-sq(pred)** |
| --- | --- | --- | --- |
| 1.43147 | 92.16% | 90.30% | 87.75% |

GS

**One-way ANOVA: Gs versus Treatments**

**Method**

| Null hypothesis | All means are equal |
| --- | --- |
| Alternative hypothesis | Not all means are equal |
| Significance level | α = 0.05 |

*Equal variances were assumed for the analysis.*

**Factor Information**

| **Factor** | **Levels** | **Values** |
| --- | --- | --- |
| Treatments | 19 | AMp1, AMp2, AMp3, C, Co1, Co2, Co3, Mp1, Mp1+Co1, Mp1+Co2, Mp1+Co3, Mp2, Mp2+Co1, Mp2+Co2, Mp2+Co3, Mp3, Mp3+Co1, Mp3+Co2, Mp3+Co3 |

**Analysis of Variance**

| **Source** | **DF** | **Adj SS** | **Adj MS** | **F-Value** | **P-Value** |
| --- | --- | --- | --- | --- | --- |
| Treatments | 18 | 0.107962 | 0.005998 | 58.72 | 0.000 |
| Error | 76 | 0.007763 | 0.000102 |  |  |
| Total | 94 | 0.115725 |  |  |  |

**Model Summary**

| **S** | **R-sq** | **R-sq(adj)** | **R-sq(pred)** |
| --- | --- | --- | --- |
| 0.0101068 | 93.29% | 91.70% | 89.52% |
