# Supplementary material for: Allelopathic interactions of Carthamus oxyacantha, Macrophomina phaseolina and maize: Implications for the use of Carthamus oxyacantha as a natural disease management strategy in maize
Source: PLoS One. 2024 Oct 31;19(10):e0307082. doi: 10.1371/journal.pone.0307082 (PMC11527155; doi:10.1371/journal.pone.0307082)
Supplement: S6 File — (DOCX) [file pone.0307082.s006.docx]

**S6. ANOVA file for the effect of treatments on (A) transpiration rate and (B) internal carbon dioxide concentration of maize.**

E (A)

**One-way ANOVA: E versus Treatments**

**Method**

| Null hypothesis | All means are equal |
| --- | --- |
| Alternative hypothesis | Not all means are equal |
| Significance level | α = 0.05 |

*Equal variances were assumed for the analysis.*

**Factor Information**

| **Factor** | **Levels** | **Values** |
| --- | --- | --- |
| Trearment | 19 | AMp1, AMp2, AMp3, C, Co1, Co2, Co3, Mp1, Mp1+Co1, Mp1+Co2, Mp1+Co3, Mp2, Mp2+Co1, Mp2+Co2, Mp2+Co3, Mp3, Mp3+Co1, Mp3+Co2, Mp3+Co3 |

**Analysis of Variance**

| **Source** | **DF** | **Adj SS** | **Adj MS** | **F-Value** | **P-Value** |
| --- | --- | --- | --- | --- | --- |
| Trearment | 18 | 10.5848 | 0.588042 | 67.27 | 0.000 |
| Error | 76 | 0.6644 | 0.008742 |  |  |
| Total | 94 | 11.2491 |  |  |  |

**Model Summary**

| **S** | **R-sq** | **R-sq(adj)** | **R-sq(pred)** |
| --- | --- | --- | --- |
| 0.0934964 | 94.09% | 92.70% | 90.77% |

CI (B)

**One-way ANOVA: Ci versus Treatments**

**Method**

| Null hypothesis | All means are equal |
| --- | --- |
| Alternative hypothesis | Not all means are equal |
| Significance level | α = 0.05 |

*Equal variances were assumed for the analysis.*

**Factor Information**

| **Factor** | **Levels** | **Values** |
| --- | --- | --- |
| Treatments | 19 | AMp1, AMp2, AMp3, C, Co1, Co2, Co3, Mp1, Mp1+Co1, Mp1+Co2, Mp1+Co3, Mp2, Mp2+Co1, Mp2+Co2, Mp2+Co3, Mp3, Mp3+Co1, Mp3+Co2, Mp3+Co3 |

**Analysis of Variance**

| **Source** | **DF** | **Adj SS** | **Adj MS** | **F-Value** | **P-Value** |
| --- | --- | --- | --- | --- | --- |
| Treatments | 18 | 100292 | 5571.8 | 50.57 | 0.000 |
| Error | 76 | 8373 | 110.2 |  |  |
| Total | 94 | 108665 |  |  |  |

**Model Summary**

| **S** | **R-sq** | **R-sq(adj)** | **R-sq(pred)** |
| --- | --- | --- | --- |
| 10.4964 | 92.29% | 90.47% | 87.96% |
