# Supplementary material for: Allelopathic interactions of Carthamus oxyacantha, Macrophomina phaseolina and maize: Implications for the use of Carthamus oxyacantha as a natural disease management strategy in maize
Source: PLoS One. 2024 Oct 31;19(10):e0307082. doi: 10.1371/journal.pone.0307082 (PMC11527155; doi:10.1371/journal.pone.0307082)
Supplement: S7 File — (DOCX) [file pone.0307082.s007.docx]

**S7. ANOVA file for the effect of treatments on (A) superoxide dismutase, (B) peroxidase, and (C) catalase activities of maize.**

SOD (A)

**One-way ANOVA: SOD versus Treatments**

**Method**

| Null hypothesis | All means are equal |
| --- | --- |
| Alternative hypothesis | Not all means are equal |
| Significance level | α = 0.05 |

*Equal variances were assumed for the analysis.*

**Factor Information**

| **Factor** | **Levels** | **Values** |
| --- | --- | --- |
| Treatments | 19 | AMp1, AMp2, AMp3, C, Co1, Co2, Co3, Mp1, Mp1+Co1, Mp1+Co2, Mp1+Co3, Mp2, Mp2+Co1, Mp2+Co2, Mp2+Co3, Mp3, Mp3+Co1, Mp3+Co2, Mp3+Co3 |

**Analysis of Variance**

| **Source** | **DF** | **Seq SS** | **Contribution** | **Adj SS** | **Adj MS** | **F-Value** | **P-Value** |
| --- | --- | --- | --- | --- | --- | --- | --- |
| Treatments | 18 | 26865 | 94.54% | 26865 | 1492.52 | 73.07 | 0.000 |
| Error | 76 | 1552 | 5.46% | 1552 | 20.43 |  |  |
| Total | 94 | 28418 | 100.00% |  |  |  |  |

**Model Summary**

| **S** | **R-sq** | **R-sq(adj)** | **PRESS** | **R-sq(pred)** |
| --- | --- | --- | --- | --- |
| 4.51964 | 94.54% | 93.24% | 2425.72 | 91.46% |

POD (B)

**One-way ANOVA: POD versus Treatments**

**Method**

| Null hypothesis | All means are equal |
| --- | --- |
| Alternative hypothesis | Not all means are equal |
| Significance level | α = 0.05 |

*Equal variances were assumed for the analysis.*

**Factor Information**

| **Factor** | **Levels** | **Values** |
| --- | --- | --- |
| Treatments | 19 | AMp1, AMp2, AMp3, C, Co1, Co2, Co3, Mp1, Mp1+Co1, Mp1+Co2, Mp1+Co3, Mp2, Mp2+Co1, Mp2+Co2, Mp2+Co3, Mp3, Mp3+Co1, Mp3+Co2, Mp3+Co3 |

**Analysis of Variance**

| **Source** | **DF** | **Seq SS** | **Contribution** | **Adj SS** | **Adj MS** | **F-Value** | **P-Value** |
| --- | --- | --- | --- | --- | --- | --- | --- |
| Treatments | 18 | 29930 | 83.66% | 29930 | 1662.78 | 21.62 | 0.000 |
| Error | 76 | 5845 | 16.34% | 5845 | 76.91 |  |  |
| Total | 94 | 35775 | 100.00% |  |  |  |  |

**Model Summary**

| **S** | **R-sq** | **R-sq(adj)** | **PRESS** | **R-sq(pred)** |
| --- | --- | --- | --- | --- |
| 8.76956 | 83.66% | 79.79% | 9132.5 | 74.47% |

CAT (C)

**One-way ANOVA: CAT versus Treatments**

**Method**

| Null hypothesis | All means are equal |
| --- | --- |
| Alternative hypothesis | Not all means are equal |
| Significance level | α = 0.05 |

*Equal variances were assumed for the analysis.*

**Factor Information**

| **Factor** | **Levels** | **Values** |
| --- | --- | --- |
| Treatments | 19 | AMp1, AMp2, AMp3, C, Co1, Co2, Co3, Mp1, Mp1+Co1, Mp1+Co2, Mp1+Co3, Mp2, Mp2+Co1, Mp2+Co2, Mp2+Co3, Mp3, Mp3+Co1, Mp3+Co2, Mp3+Co3 |

**Analysis of Variance**

| **Source** | **DF** | **Seq SS** | **Contribution** | **Adj SS** | **Adj MS** | **F-Value** | **P-Value** |
| --- | --- | --- | --- | --- | --- | --- | --- |
| Treatments | 18 | 38.475 | 87.94% | 38.475 | 2.13749 | 30.80 | 0.000 |
| Error | 76 | 5.275 | 12.06% | 5.275 | 0.06940 |  |  |
| Total | 94 | 43.749 | 100.00% |  |  |  |  |

**Model Summary**

| **S** | **R-sq** | **R-sq(adj)** | **PRESS** | **R-sq(pred)** |
| --- | --- | --- | --- | --- |
| 0.263445 | 87.94% | 85.09% | 8.24167 | 81.16% |
