# Supplementary material for: Allelopathic interactions of Carthamus oxyacantha, Macrophomina phaseolina and maize: Implications for the use of Carthamus oxyacantha as a natural disease management strategy in maize
Source: PLoS One. 2024 Oct 31;19(10):e0307082. doi: 10.1371/journal.pone.0307082 (PMC11527155; doi:10.1371/journal.pone.0307082)
Supplement: S1 Table — (DOCX) [file pone.0307082.s008.docx]

**S8 Table. Values used for Principal Component Analysis (PCA).**

| Treatments | **SL** | **SDM** | **RDM** | **Chl *a*** | **Chl *b*** | **Carotenoid** | ***A*** | **g*s*** | ***E*** | **C*i*** | **SOD** | **POD** | **CAT** | **AUDIPC** | **AUDSPC** |
| --- | --- | --- | --- | --- | --- | --- | --- | --- | --- | --- | --- | --- | --- | --- | --- |
| **C** | 97 | 13.7 | 2.3 | 9.8 | 5.8 | 155 | 25.8 | 0.189 | 1.90 | 176 | 41.39 | 92.8 | 1.44 | 0.00 | 0 |
| **Mp1** | 72 | 10.1 | 1.6 | 6.8 | 3.6 | 112 | 18.5 | 0.135 | 1.52 | 200 | 54.61 | 101.2 | 2.12 | 1820.00 | 756 |
| **Mp2** | 68 | 8.4 | 1.3 | 6.1 | 2.9 | 101 | 17 | 0.127 | 1.40 | 220.4 | 64.29 | 108.8 | 2.40 | 2240.00 | 1400 |
| **Mp3** | 65 | 7.1 | 1.1 | 5.8 | 2.6 | 94 | 13.8 | 0.103 | 1.18 | 232.2 | 74.28 | 115.6 | 2.80 | 2800.00 | 1960 |
| **AMp1** | 92 | 13.4 | 2.2 | 9.3 | 5.5 | 152 | 24 | 0.182 | 1.76 | 172 | 45.36 | 93.6 | 1.56 | 0.00 | 0 |
| **AMp2** | 87 | 12.7 | 2.1 | 9.0 | 5.3 | 143 | 23.5 | 0.175 | 1.75 | 167.8 | 48.73 | 96.4 | 1.73 | 0.00 | 0 |
| **AMp3** | 83 | 12.4 | 2 | 8.3 | 4.9 | 139 | 22.8 | 0.168 | 1.67 | 158.8 | 54.31 | 101.2 | 1.84 | 0.00 | 0 |
| **CO1** | 100 | 14.8 | 2.6 | 11.9 | 6.2 | 162 | 26.4 | 0.192 | 2.00 | 195 | 46.97 | 94.4 | 1.96 | 0.00 | 0 |
| **CO2** | 108 | 16.6 | 2.7 | 12.4 | 6.5 | 166 | 28.4 | 0.221 | 2.15 | 206.2 | 51.82 | 97.6 | 2.11 | 0.00 | 0 |
| **CO3** | 111 | 17.3 | 3 | 12.7 | 6.7 | 172 | 29.9 | 0.237 | 2.43 | 212.4 | 57.54 | 105.2 | 2.35 | 0.00 | 0 |
| **Mp1+CO1** | 97 | 13.5 | 2.4 | 9.9 | 5.2 | 152 | 24.5 | 0.172 | 1.65 | 230.6 | 59.74 | 118.8 | 2.65 | 1540.00 | 644 |
| **Mp1+CO2** | 103 | 14.4 | 2.6 | 10.4 | 5.7 | 158 | 26.8 | 0.194 | 1.89 | 237 | 72.37 | 127.2 | 2.88 | 980.00 | 448 |
| **Mp1+CO3** | 107 | 16.7 | 2.8 | 10.8 | 5.9 | 164 | 28.4 | 0.212 | 2.24 | 240.6 | 76.77 | 135.6 | 3.17 | 560.00 | 308 |
| **Mp2+CO1** | 87 | 10.8 | 1.9 | 8.6 | 4.3 | 126 | 21.3 | 0.157 | 1.46 | 245.4 | 73.98 | 122 | 2.77 | 1820.00 | 896 |
| **Mp2+CO2** | 93 | 11.7 | 2 | 9.1 | 4.5 | 131 | 23.5 | 0.176 | 1.65 | 251.4 | 87.93 | 130 | 3.23 | 1260.00 | 672 |
| **Mp2+CO3** | 96 | 12.9 | 2.2 | 9.6 | 4.7 | 136 | 25.5 | 0.188 | 2.04 | 256.2 | 90.28 | 140 | 3.36 | 700.00 | 392 |
| **Mp3+CO1** | 76 | 8.9 | 1.5 | 7.4 | 3.6 | 113 | 16.8 | 0.126 | 1.22 | 249 | 81.47 | 132 | 2.97 | 2240.00 | 1232 |
| **Mp3+CO2** | 80 | 10.2 | 1.6 | 8.3 | 3.8 | 120 | 18.5 | 0.142 | 1.34 | 256 | 92.92 | 138.8 | 3.28 | 1820.00 | 868 |
| **Mp3+CO3** | 84 | 11.1 | 1.8 | 8.6 | 4.1 | 126 | 19.3 | 0.153 | 1.70 | 265 | 94.09 | 148.4 | 3.59 | 1260.00 | 756 |
