# Supplementary material for: Allelopathic interactions of Carthamus oxyacantha, Macrophomina phaseolina and maize: Implications for the use of Carthamus oxyacantha as a natural disease management strategy in maize
Source: PLoS One. 2024 Oct 31;19(10):e0307082. doi: 10.1371/journal.pone.0307082 (PMC11527155; doi:10.1371/journal.pone.0307082)
Supplement: Attachment — Submitted filename: PONE-D-23-34629.pdf [file pone.0307082.s009.pdf]

PLOS ONE

Carthamus oxyacantha as bio-elicitor and natural defense in maize against charcoal rot disease caused by Macrophomina phaseolina

--Manuscript Draft--

Manuscript Number:	PONE-D-23-34629
Article Type:	Research Article
Full Title:	Carthamus oxyacantha as bio-elicitor and natural defense in maize against charcoal rot disease caused by Macrophomina phaseolina
Short Title:	Antifungal efficacy of Carthamus oxyacantha against Macrophomina phaseolina
Corresponding Author:	Muhammad Akbar, PhD<br>University of Gujrat<br>Gujrat, Punjab PAKISTAN
Keywords:	Carthamus oxyacantha, charcoal rot, Antifungal activity, bio-elicitor, maize
Abstract:	Fungicides are used to control phytopathogen but all these fungicides have deleterious effects. In the present study, the antifungal efficacy of Carthamus oxyacantha was assessed against Macrophomina phaseolina, the cause of charcoal rot in maize. Total 19 treatments were investigated in pot experiment. These treatments included a negative control, 3 concentrations of inoculum (1.2×105 mL-1, 2.4×105 CFU mL-1 and 3.6×105 CFU mL-1), and w/w 3, concentrations (0.5, 1.0 and 1.5%) of C. oxyacantha. The treatment with dead inoculum and C. oxyacantha alone were included to investigate their allelopathic effect on maize, not investigated earlier. Maximum suppression of disease was observed by 1.5% (w/w) conc. of C. oxyacantha. Soil amendment with dry powder of C. oxyacantha suppressed disease incidence (DI) and disease severity index (DSI) in charcoal rot of maize significantly up to 40 and 55%, respectively over the strongest level of inoculum (Mp3). C. oxyacantha not only reduced area under disease incidence progress curve (AUDIPC), area under disease severity curve (AUDSPC), but also improved the morphological, biochemical and physiological parameters of maize. Maximum increase of 48.1, 65.3, and 75% in the values of shoot length (SL), shoot dry mass (SDM), and root dry mass (RDM) respectively was observed by application of the highest conc. of Carthamus powder in the treatment Mp1+Co3, over infested control (Mp1). Photosynthetic pigments, such as Chlorophyll a, Chlorophyll b and carotenoids were increased to 58, 64, and 46%, respectively over Mp1, by the application of Carthamus powder. Carbon assimilation rate (A), stomatal conductance (gs), rate of transpiration (E), and internal carbon dioxide concentration (Ci) were significantly increased to 58, 48, 48, and 20% respectively over infested control (Mp3), by application of Carthamus powder @ 1.5 (w/w) conc. Moreover, defense enzymes (SOD, POD and CAT) activities were boost up to 26.7, 28.4, and 28.1% over Mp3, respectively.
Order of Authors:	Nazir Aslam, PhD Scholar
	Muhammad Akbar, PhD
Additional Information:	
Question	Response
**Financial Disclosure**<br><br>Enter a financial disclosure statement that describes the sources of funding for the work included in this submission. Review the submission guidelines for detailed requirements. View published research articles from *PLOS ONE* for specific examples.	The author(s) received no specific funding for this work.

Powered by Editorial Manager® and ProduXion Manager® from Aries Systems Corporation

This statement is required for submission and **will appear in the published article** if the submission is accepted. Please make sure it is accurate.<br><br>**Funded studies**<br>Enter a statement with the following details:<br>• Initials of the authors who received each award<br>• Grant numbers awarded to each author<br>• The full name of each funder<br>• URL of each funder website<br>• Did the sponsors or funders play any role in the study design, data collection and analysis, decision to publish, or preparation of the manuscript?<br><br>Did you receive funding for this work?	
**Competing Interests**<br><br>Use the instructions below to enter a competing interest statement for this submission. On behalf of all authors, disclose any competing interests that could be perceived to bias this work—acknowledging all financial support and any other relevant financial or non-financial competing interests.<br><br>This statement is required for submission and **will appear in the published article** if the submission is accepted. Please make sure it is accurate and that any funding sources listed in your Funding Information later in the submission form are also declared in your Financial Disclosure statement.<br><br>View published research articles from *PLOS ONE* for specific examples.	The authors have declared that no competing interests exist.

**NO authors have competing interests**<br><br>Enter: *The authors have declared that no competing interests exist.*<br><br>**Authors with competing interests**<br><br>Enter competing interest details beginning with this statement:<br><br>*I have read the journal's policy and the authors of this manuscript have the following competing interests: [insert competing interests here]*<br><br>* typeset	
**Ethics Statement**<br><br>Enter an ethics statement for this submission. This statement is required if the study involved:<br><br>• Human participants<br>• Human specimens or tissue<br>• Vertebrate animals or cephalopods<br>• Vertebrate embryos or tissues<br>• Field research<br><br>Write "N/A" if the submission does not require an ethics statement.<br><br>General guidance is provided below. Consult the submission guidelines for detailed instructions. **Make sure that all information entered here is included in the Methods section of the manuscript.**	N/A

Powered by Editorial Manager® and ProduXion Manager® from Aries Systems Corporation

**Format for specific study types**<br><br>**Human Subject Research (involving human participants and/or tissue)**<br>• Give the name of the institutional review board or ethics committee that approved the study<br>• Include the approval number and/or a statement indicating approval of this research<br>• Indicate the form of consent obtained (written/oral) or the reason that consent was not obtained (e.g. the data were analyzed anonymously)<br><br>**Animal Research (involving vertebrate animals, embryos or tissues)**<br>• Provide the name of the Institutional Animal Care and Use Committee (IACUC) or other relevant ethics board that reviewed the study protocol, and indicate whether they approved this research or granted a formal waiver of ethical approval<br>• Include an approval number if one was obtained<br>• If the study involved *non-human primates*, add *additional details* about animal welfare and steps taken to ameliorate suffering<br>• If anesthesia, euthanasia, or any kind of animal sacrifice is part of the study, include briefly which substances and/or methods were applied<br><br>**Field Research**<br><br>Include the following details if this study involves the collection of plant, animal, or other materials from a natural setting:<br>• Field permit number<br>• Name of the institution or relevant body that granted permission	
**Data Availability**<br><br>Authors are required to make all data underlying the findings described fully available, without restriction, and from the time of publication. PLOS allows rare exceptions to address legal and ethical concerns. See the PLOS Data Policy and FAQ for detailed information.	Yes - all data are fully available without restriction

A Data Availability Statement describing where the data can be found is required at submission. Your answers to this question constitute the Data Availability Statement and **will be published in the article**, if accepted.<br><br>**Important:** Stating 'data available on request from the author' is not sufficient. If your data are only available upon request, select 'No' for the first question and explain your exceptional situation in the text box.<br><br>Do the authors confirm that all data underlying the findings described in their manuscript are fully available without restriction?	
**Describe where the data may be found in full sentences. If you are copying our sample text, replace any instances of XXX with the appropriate details.**<br><br>• If the data are **held or will be held in a public repository**, include URLs, accession numbers or DOIs. If this information will only be available after acceptance, indicate this by ticking the box below. For example: *All XXX files are available from the XXX database (accession number(s) XXX, XXX.).*<br>• If the data are all contained **within the manuscript and/or Supporting Information files**, enter the following: *All relevant data are within the manuscript and its Supporting Information files.*<br>• If neither of these applies but you are able to provide **details of access elsewhere**, with or without limitations, please do so. For example:<br><br>*Data cannot be shared publicly because of [XXX]. Data are available from the XXX Institutional Data Access / Ethics Committee (contact via XXX) for researchers who meet the criteria for access to confidential data.*<br><br>*The data underlying the results presented in the study are available from (include the name of the third party*	No

and contact information or URL).<br>• This text is appropriate if the data are owned by a third party and authors do not have permission to share the data.<br><br>* typeset	
Additional data availability information:	Tick here if the URLs/accession numbers/DOIs will be available only after acceptance of the manuscript for publication so that we can ensure their inclusion before publication.

Powered by Editorial Manager® and ProduXion Manager® from Aries Systems Corporation

Manuscript
Click here to access/download;Manuscript;Manuscript.docx

Carthamus oxyacantha **as bio-elicito[illegible]nd natural defense in maize against charcoal rot disease caused by *Macrophomina phaseolina***

Nazir Aslam[1], Muhammad Akbar[1]*

[1]Department of Botany, University of Gujrat, Gujrat, 50700, Punjab, Pakistan.

Submit correspondence to: Muhammad Akbar at the address provided. E-mail: muhammad.akbar@uog.edu.pk

Abstract

Fungicides are used to control phytopathogen but all these fungicides have deleterious effects. In the present study, the antifungal efficacy of *Carthamus oxyacantha* was assessed against *Macrophomina phaseolina*, the cause of charcoal rot in maize. Total 19 treatments were investigated in pot experiment. These treatments included a negative control, 3 concentrations of inoculum (1.2×10^5 mL^{-1}, 2.4×10^5 CFU mL^{-1} and 3.6×10^5 CFU mL^{-1}), and w/w 3, concentrations (0.5, 1.0 and 1.5%) of *C. oxyacantha*. The treatment with dead inoculum and *C. oxyacantha* alone were included to investigate their allelopathic effect on maize, not investigated earlier. Maximum suppression of disease was observed by 1.5% (w/w) conc. of *C. oxyacantha.* Soil amendment with dry powder of *C. oxyacantha* suppressed disease incidence (DI) and disease severity index (DSI) in charcoal rot of maize significantly up to 40 and 55%, respectively over the strongest level of inoculum (Mp3). C. *oxyacantha* not only reduced area under disease incidence progress curve (AUDIPC), area under disease severity curve (AUDSPC), but also improved the morphological, biochemical and physiological parameters of maize. Maximum increase of 48.1, 65.3, and 75% in the values of shoot length (SL), shoot dry mass (SDM), and root dry mass (RDM) respectively was observed by application of the highest conc. of *Carthamus* powder in the treatment Mp1+Co3, over infested control

(Mp1). Photosynthetic pigments, such as Chlorophyll a, Chlorophyll b and carotenoids were increased to 58, 64, and 46%, respectively over Mp1, by the application of *Carthamus* powder. Carbon assimilation rate (*A*), stomatal conductance (*gs*), rate of transpiration (*E*), and internal carbon dioxide concentration (*Ci*) were significantly increased to 58, 48, 48, and 20% respectively over infested control (Mp3), by application of *Carthamus* powder @ 1.5 (w/w) conc. Moreover, defense enzymes (SOD, POD and CAT) activities were boost up to 26.7, 28.4, and 28.1% over Mp3, respectively.

Key words: *Carthamus oxyacantha,* charcoal rot, Antifungal activity, bio-elicitor, maize

1. Introduction

Maize (*Zea mays* L.) is a staple food crop which stands at 1st position with respect to its production, while it stands at 3rd position regarding its cultivation worldwide, after wheat and rice [1]. Pakistan is on 18th number among the maize producing countries, with its 5.27 t ha^{-1} yield, that is very low as compared to per hectare yield, 9.5 t ha^{-1} in United States [2,3]. There are many factors involved in per hectare low yield, including non-availability of high yielding maize varieties, costly fertilizers, poor implementation of agricultural policies, adverse climatic conditions, and biotic constraints [4].

Pathogenic fungi are the main biotic constraints regarding plant diseases and annually 31% loss in maize yield has been recorded [5]. Among them *Macrophomina phaseolina* (Tassi) Goid, a seed and soil borne, anamorphic and necrotrophic pathogen, infects over 500 hosts with morphological symptoms such as, charcoal rot, canker, damping off and blights in some crops [6].

Charcoal rot of maize has developed into more alarming situation because of climatic resistance found in pathogen, epidemic nature and its propensity to kill the host plants, causing up to 63.6% yield loss in maize crop [7,8]. *M. phaseolina* is a soil and seed born pathogen, its transmission from seed to seedling has also been reported [9]. Pakistan is one among the countries which are facing devastating environmental changes. Rise in average daily temperature of Pakistan, up to 0.87 ºC is slightly more than average global increase. Meanwhile, Pakistan experiences some of the highest maximum temperatures in the world, with average

maximum temperature of 38 °C and above in many regions [10]. So, this climate change is very favorite for *M. phaseolina,* causal agent of charcoal rot.

Various strategies such as avoiding, breeding for disease resistance, cultural disease management strategies and chemical disease management can be adopted to control charcoal rot of maize. However, presence of natural antifungal compounds in plants is an emerging technology to uplift economics in crops by managing plant diseases [11, 12, 13, 14]. Several successful investigations reported that application of plant extracts, residue, compost and mulches used either *in-vitro* or *in-vivo* significantly controlled various fungal pathogens [15,16, 17, 18]. Previous *in vitro* studies revealed, *C. oxyacantha* has antifungal activity against various fungal pathogens such as *Aspergillus niger*, *M. phaseolina*, *Rhizoctonia solani*, *Fusarium oxysporum,* and mushrooms [19, 20]. It also contains antifungal compounds [21, 22]. Along with presence of antifungal compounds in an organic material to cope fungal diseases in plants, its compatibility with crop plants is also very important. Allelopathy of plant residue must be exploited before its application in field [23]. Although, *in vitro* antifungal activity of *C. oxyacantha* has been reported, there effectiveness has never been tested against charcoal rot of maize in *in-vivo* as well as its compatibility for maize in pots were missing also. Therefore, pot experiment was arranged to investigate compatibility, antifungal efficacy of *C. oxyacantha*, to control charcoal rot of maize caused by *M. phaseolina*. Moreover, effect of soil amendment with dry powder of *C. oxyacantha*, on morphological, physiological, and defense related attributes of maize were also investigated 1st time in this study.

2. Materials and methods

2.1 Collection of indigenous weed, pathogen and test plant

Indigenous weed, *Carthamus oxyacantha* was collected from District Mandi Bahauddin, Punjab Province, Pakistan, and identified by Dr. Muhammad Akbar, Associate Professor in the Department of Botany at University of Gujrat, Pakistan. The plants were air-dried under shade. Dried plants were grinded in to powder with the help of mini electric grinder Nm-8300, and stored at room temperature, in polythene bags.

Certified seeds of maize, variety Neelam, were purchased from the local market. The isolate of fungal pathogen, *M. phaseolina* was isolated from diseased maize plant collected from maize field, village Sagera, District Kasur, Punjab, Pakistan. Sorghum seeds were used as substrate for multiplication of fungal inoculum [24].

2.2 *In vivo* assessment of antifungal activity of weed powder

Antifungal efficacy of weed powder was investigated in a pot experiment (Pots were kept in open air to stimulate natural field conditions. Whereas, pots were used to enhance inoculum equally for equable infection and better control of weed powder) with a completely randomized design (CRD), having 19 treatments with 5 replicates. Corn field soil and pots (20 cm in diameter and 30 cm deep) were sterilized by 5% formalin solution for 15 minutes and left to dry for two weeks. Soil amendments with selected weed powder were made with three w/w concentrations (0.5, 1.0 and 1.5%) by mixing the weed powder in pots of selected treatments. Amended soil was added in each pot (7 kg/pot). Sorghum based fungal inoculum [25] was grounded to form hyphal/microsclerotial suspension in sterilized distilled water and three concentrations 1.2×10^5 mL^{-1}, 2.4×10^5 CFU mL^{-1} and 3.6×10^5 CFU mL^{-1}, respectively were maintained by serial dilution method. Pot soil of selected treatments was infested by inoculum of the pathogen by mixing ten ml of hyphal/microsclerotia suspension in topsoil of 10 cm depth. Healthy maize seeds were sown on 5th day of inoculation [26], at rate 3 seeds/pot and each pot was thinned to one seedling at ten days after emergence. Standard agronomical conditions were maintained [27]. In total 19 treatments were made. The detailed composition of treatments in pot experiment is given in the table 1.

Table 1: Treatments composition in pot experiment

S.N	Treatments	Description
**1**	C	Control (Without pathogen and soil amendment)
**2**	Mp1	*Macrophomina phaseolina* (1.2×10^{5} CFU mL^{-1})
**3**	Mp2	*Macrophomina phaseolina* (2.4×10^{5} CFU mL^{-1})
**4**	Mp3	*Macrophomina phaseolina* (3.6×10^{5} CFU mL^{-1})
**5**	AMp1	Autoclaved *Macrophomina phaseolina* (1.2×10^{5} CFU mL^{-1})
**6**	AMp2	Autoclaved *Macrophomina phaseolina* (2.4×10^{5} CFU mL^{-1})
**7**	AMp3	Autoclaved *Macrophomina phaseolina* (3.6×10^{5} CFU mL^{-1})
**8**	Co1	*Cathamus oxyacantha* 0.5% (w/w)
**9**	Co2	*Cathamus oxyacantha* 1% (w/w)
**10**	Co3	*Cathamus oxyacantha* 1.5% (w/w)
**11**	Mp1+Co1	*Macrophomina phaseolina* (1.2×10^{5} CFU mL^{-1})+ *Cathamus oxyacantha* 0.5%
**12**	Mp1+Co2	*Macrophomina phaseolina* (1.2×10^{5} CFU mL^{-1})+ *Cathamus oxyacantha* 1%
**13**	Mp1+Co3	*Macrophomina phaseolina* (1.2×10^{5} CFU mL^{-1})+ *Cathamus oxyacantha* 1.5%
**14**	Mp2+Co1	*Macrophomina phaseolina* (2.4×10^{5} CFU mL^{-1})+ *Cathamus oxyacantha* 0.5%
**15**	Mp2+Co2	*Macrophomina phaseolina* (2.4×10^{5} CFU mL^{-1})+ *Cathamus oxyacantha* 1%
**16**	Mp2+Co3	*Macrophomina phaseolina* (2.4×10^{5} CFU mL^{-1})+ *Cathamus oxyacantha* 1.5%
**17**	Mp3+Co1	*Macrophomina phaseolina* (3.6×10^{5} CFU mL^{-1})+ *Cathamus oxyacantha* 0.5%
**18**	Mp3+Co2	*Macrophomina phaseolina* (3.6×10^{5} CFU mL^{-1})+ *Cathamus oxyacantha* 1%
**19**	Mp3+Co3	*Macrophomina phaseolina* (3.6×10^{5} CFU mL^{-1})+ *Cathamus oxyacantha* 1.5%

2.3 Disease assessment

Disease incidence (DI) and disease severity index (DSI) were assessed simultaneously, three times in whole pot experiments with the interval of 14 days. Disease symptoms for charcoal rot of maize, appearance of lesion at the collar region [26] were observed after 42 days after sowing (DAS), at growth stage 3, (Collar of 12th leaf visible, leaves 3 and 4 may be dead) in plants of infested pots. Two more successive observations were made with the interval of 14 days, at growth stage 4, 56 DAS (Collar of 14th leaves visible, tips of many tassels visible) and at growth stage between 5 & 6, 70 DAS (75% of plants have silks visible) [28]. Both DI and DSI were evaluated at the same time. Depending on the disease symptoms, DSI was scaled on a 0–5 scale [29]. Numerical disease rating was assigned as follows: 0, healthy plants; 1, appearance of lesion at the collar region, 2–7 mm in length; 2, large lesions, 8- 12 mm in length; 3, moderate rotting of the collar region, loss of turgor at the top with slight drooping; 4, extensive rotting at the collar region, wilting and drying of many leaves, drooping of the shoot; 5, plants completely wilted, dead and dry. DI and DSI were measured by following equations.

$$\mathrm{DI} = \frac{\text{No. of diseased plants}}{\text{Total number of Plants}} \times 100 \quad (1)$$

$$\mathrm{DSI} = \frac{0\times P0+1\times P1+2\times P2+3\times P3+4\times P4+5\times P5}{N(G-1)} \times 100 \quad (2)$$

Where P0 to P5 are total number of observed plants in each disease grading per treatment and N is total number of observations [30]. The area under disease incidence progress curve (AUDIPC) and area under disease severity progress curve (AUDSPC) both were calculated by [30].

$$\mathrm{AUDIPC/AUDSPC} = \sum_{n=1}^{n-1} (X_i + Xi - 1/2)(\mathrm{T}i - \mathrm{T}i - 1) \quad (3)$$

Where Xi is 1st reading of disease incidence/disease severity and xi+1 indicated each successive reading of disease incidence/disease severity at time (t).

2.4 Morphological parameters

Morphological parameters shoot length was measured by measuring tap, shoot dry weight and root dry weight of all plants of all treatments were recorded after oven drying at 70 ºC, till constant dry weight reading, after harvesting on 80 DAS [31].

2.5 Estimation of photosynthetic pigments

The measurement of photosynthetic pigments such as chlorophyll a, b, and carotenoids was done according to Arnon (1949) at 42 DAS. For this, 100 mg of apical leaves from each treatment (parts of three leaves were randomly mixed) were cut into small pieces and mixed with 5 mL of 80% acetone in triplicates. Homogenization was done in a pre-cooled sterile mortar and pestle. The obtained extract was then centrifuged at 3000 rpm for 15 min and the clear solution was transferred to a new vial with a final volume made up to 5 mL by 80 % acetone. For these three pigments, the optical density was recorded at 663, 645, and 440.5 nm wavelength, respectively [32]. The levels of chlorophyll a, b and carotenoids were measured by the following equations;

$$Chlorophyll\ a\ mg\ \mathrm{g}^{-1} = 12.7\ (OD)\ 663 – 2.69 (OD) 645 \times (w/v \times 1000) \quad (4)$$

$$Chlorophyll\ b\ mg\ g^{-1} = 22.9\ (OD)\ 645 - 4.68\ (OD)\ 663 \times (w/v \times 1000) \quad (5)$$

$$Total\ carotenoids\ mg\ \mathrm{g}^{-1} = 46.95\ (OD)\ 440.5 - (0.268 \times chl\ a + b) \quad (6)$$

where v = final volume (ml) of extract in 80% acetone, w = fresh weight of leaf in grams

2.6 Physiological measurements

Physiological measurements were made after 56 DAS on the 6th leaf of plants from each treatment (5 replications per treatment) with help of a portable infrared gas analyzer (IRGA) (model: ADC-USA 1264) and following four parameters were calculated: Net carbon assimilation rate (A) µ mol $CO_2m\text{-}^2s^{-1}$; Stomatal conductance (*gs*) mmolm-$^2s^{-1}$; Internal CO_2 concentration (*Ci*); Transpiration rate (*E*) mmol$H_2Om\text{-}^2s^{-1}$. All the measurements were made from 10:00 am to 2:00 pm.

2.7 Measurements of antioxidant activities

For SOD, POD and CAT antioxidant activities 0.5 g of fresh leaf tissues (mixed from 10 leaves collected from plant for each treatment at 56 DAS were ground into a fine powder by pre chilled mortar and pestle. Homogenized the leaf powder by adding 3 ml of chilled 100 mM PBS buffer (pH 7.8) [33]. After adding 1.5mL of homogenate in two centrifuge tubes, centrifuged at 10,000 *x g* for 20 min at 4 °C. Centrifuged supernatant was transferred to new centrifuge tubes for further analysis.

2.7.1 SOD activity assay

Solution mixture (for 95 reactions) were prepared by adding 95 mL 100 mM PBS (pH 7.8), 1.9 mL 1 mM EDTA, 6.4 mL 130 mM Met, 6.4 mL 750 µM NBT, and 6.4 mL 20 µM Riboflavin. Crude enzyme solution (50 µL) crude enzyme from each sample was added into 1 mL reaction solution in a 1.5 ml centrifuge tube. Reaction solution with 50 µL 100 mM PBS (pH 7.8) but no crude enzyme under dark and light condition served as controls I and control II, respectively. Put the tubes as well as control II under light, uniformly, with the light intensity of 4,000 lux for 10-15 min. By contrast, kept control I in the dark and moved the other tubes away from the light quickly. Spectrophotometer absorbance readings were measured at 560 nm in the dark and used control I as reference [34].

$$SOD\ total\ activity\ (unit\text{: } u/gFW)\ =\ [(AC - As)\ x\ V]\ /\ (0.5\ x\ Ack\ x\ Vt)/FW \qquad (7)$$

A_C: Control II, absorbance at 560 nm, A_S: sample tube, absorbance at 560 nm, V: total volume of enzyme solution, Vt: volume of enzyme used in the testing tube, FW: fresh weight of sample (g).

2.7.2 POD activity assay

Solution mixture (for 95 reactions) was prepared by adding 53.2 µL 0.2% guaiacol in 95 mL 100 mM PBS (pH 7.0), heat and stir well, add 36.1 µL 30% H_2O_2 after cooling. 50 µL 100 mM PBS (pH 7.8) and 1 mL of the reaction solution were mixed into a cuvette for reference (control) [34, 35].

$$POD\ activity\ (unit: u/gFW)\ = \triangle A470\ x\ (V/Vt)/(0.01\ x\ t)/FW \quad (8)$$

$\triangle$A470: the change in absorbance at 470 nm during every 20 seconds, V: total volume of enzyme solution, Vt: volume of enzyme used in cuvette, t: time of reaction (min), FW: sample fresh weight (g).

2.7.3 CAT activity assay

Solution mixture (for 95 reactions) was prepared by adding 147.25 µL 30% H_2O_2 in 95 mL100 mM Phosphate buffer solution (PBS (pH 7.0). 50 µL crude enzyme and 1 mL of the reaction solution were mixed into the cuvette and immediately absorbance at 240 nm with spectrophotometer was recorded at every 15 seconds for 1 min, by looking for steady average alteration. Reaction solution with 50 µL 100 mM PBS (pH 7.8) was served as a reference.

$$CAT\ activity\ (unit: u/mg\ protein)\ = \triangle A240\ x\ (V/Vt)/(0.1\ x\ t)/FW \quad (9)$$

$\triangle$A240: the change of absorbance at 240 nm during every 20 sec. V: total volume of crude enzyme solution, Vt: volume of crude enzyme used in the testing tube t: reaction time (min), FW: Fresh weight (g)

2.8. Statistical analysis

For statistical analysis, ANOVA was done followed by Fisher's LSD test at 5% probability using computer software Minitab 20.

3. Results

3.1 Disease assessment

Disease incidence (DI) and disease severity index (DSI) were measured at three different stages, 42 DAS, 56DAS and 70 DAS. There were no disease symptoms in negative control and treatments with only weed powder of *C. oxyacantha*. When comparing three different strengths of the inoculum, DI and DSI recorded for Mp1, Mp2 and Mp3 were increased progressively with stages from 60 up to 80% for DI and from 16 up to 88 for DSI, respectively. Soil amendments with Co1, Co2, and Co3 dry weed powder of *C. oxyacantha* significantly reduced the DI and DSI over positive controls with all tested strengths of inoculums. This antifungal efficacy of weed powder was reduced with the passage of time but increased with increasing quantity of the powder in respective treatments. Finally, at 70 DAS, among three tested concentrations of weed powder, (Mp3+Co3) suppressed DI and DSI in charcoal rot of maize significantly up to 40 and 55%, respectively over the strongest level of inoculum (Mp3). From three consecutive reading with 14 days of interval, area under disease incidence progress curve (AUDIPC) and area under disease incidence progress curve (AUDSPC) was calculated. Both AUDIPC and AUDSPC for Mp1, Mp2, and Mp3 were increased with from 1820 up to 2800% for AUDIPC and from 416 up to 1180% for AUDSPC, respectively. Finally, at 70 DAS, among three tested concentrations of weed powder, (Mp3+Co3) suppressed AUDIPC and AUDSPC in charcoal rot of maize significantly up to 55 and 65%, respectively over the strongest level of the inoculum, Mp3, (Table 2).

Table-2: Effect of different treatments on disease incidence (DI), disease severity index (DSI), area under disease incidence progress curve (AUDIPC), and area under disease severity progress curve (AUDSPC) on maize plants, grown in pots

Treatments	Disease Incidence (%)				Disease Severity Index			
	42DAS	56DAS	70DAS	AUDIPC	42DAS	56DAS	70DAS	AUDSPC
**Mp1**	60±24.49 a-c	60±24.49 a-c	80±20.00 ab	1820±474.77 a-d	16±7.5 bc	24±11.66 b-d	14±14.70 b-d	432±200.46 b-d
**Mp2**	80±20.00 ab	80±20 ab	80±20 ab	2240±408.2 ab	24±11.7 b	48±13.56 ab	80±20.00 ab	780±245.60 ab
**Mp3**	100±0.00 a	100±0.00 a	100±0.00 a	2800±0.0 a	56±11.7 a	68±4.90 a	88±4.90 a	1180±79.25 a
**Mp1+Co1**	40±24.49 b-d	60±24.49 a-c	60±24.49 a-c	1540±560.0 b-e	12±12.0 bc	24±14.70 b-d	32±13.56 c-e	364±151.45 c-e
**Mp1+Co2**	20±20.00 cd	40±24.49 b-d	40±24.49 b-d	980±610.3 c-f	8±8.0 bc	16±9.80 cd	24±14.70 de	248±158.19 de
**Mp1+Co3**	0±00.00 d	20±20.00 cd	20±20.00 cd	420±420.0 ef	4±4.0 bc	12±12.00 cd	16±16.00 de	168±135.74 e
**Mp2+Co1**	60±24.49 a-c	60±24.49 a-c	80±20.49 ab	1820±523.83 a-d	20±12.6 bc	28±12.00 bc	52±24.17 a-d	496±201.83 b-d
**Mp2+Co2**	40±24.49 b-d	40±24.49 b-d	60±.24.49 a-c	1260±641.6 b-e	12±8.0 bc	24±8.00 b-d	36±18.33 c-e	372±153.57 b-e
**Mp2+Co3**	20±20.00 cd	20±20.00 cd	40±24.49 b-d	700±542.3 d-f	4±4.0 bc	12±8.00 cd	28±17.44 c-e	192±117.58 de
**Mp3+Co1**	80±20.00 ab	80±20 ab	80±20.00 ab	2240±560.0 ab	24±9.8 b	44±11.66 ab	64±16.004 a-c	692±194.59 bc
**Mp3+Co2**	80±20.00 ab	60±24.49 a-c	80±20.00 ab	1960±560.0 a-c	16±11.7 bc	32±13.50 bc	44±11.66 b-d	488±206.46 b-d
**Mp3+Co3**	40±24.49 b-d	40±24.49 b-d	60±24.49 a-c	1260±560.0 b-e	12±8.0 bc	28±17.44 bc	40±16.73 cd	416±231.64 b-d

Mp1; *Macrophomina phaseolina* (1.2×10^5 CFU mL^{-1}), **Mp2;** *Macrophomina phaseolina* (2.4×10^5 CFU mL^{-1}), **Mp3;** *Macrophomina phaseolina* (3.6×10^5 CFU mL^{-1}), **Co1;** *Carthamus oxyacantha* 0.5%, **Co2;** *Carthamus oxyacantha* 1%, **Co3;** *Carthamus oxyacantha* 1.5%, **DAS**; days after sowing, **AUDIPC**; Area under disease incidence progress curve**, AUDSPC;** Area under disease severity progress curve. Data presented represent means ± standard error of 5 replicates, followed by a different alphabet, differ significantly at $P < 0.05$ according to Fisher's LSD test.

Note: Disease symptoms of charcoal rot did not appear in treatments *viz*., **C;** control**, AMp1;** Autoclaved *Macrophomina phaseolina* (1.2×10^5 CFU mL^{-1}), **AMp2;** Autoclaved *Macrophomina phaseolina* (2.4×10^5 CFU mL^{-1}), **AMp3;** Autoclaved *Macrophomina phaseolina* (3.6×10^5 CFU mL^{-1}) **Co1, Co2,** and **Co3.** So these data are excluded from the table.

3.2. Effects of treatments with *Carthamus oxyacantha* on morphological attributes of maize

Shoot length (SL), shoot dry mass (SDM), and root dry mass (RDM) were significantly decreased by the application of three different inoculum levels (Mp1, Mp2, and Mp3), up to (24.6, 29, and 32.3%), (26.3, 38.7, and 48.2%), and (30.4, 43.5, and 52.2%), respectively, over control (C). Whereas, soil amendment with powder of *C. oxyacantha* with three conc. (Co1, Co2, and Co3) increased SL, SDM, and RDM up to (4.2, 12, and 15.2%), (8, 21.2, and 26.3%), and (13, 17.4, and 30.4%), respectively over C. Application of *Carthamus* powder in infected treatments also significantly increased SL, SDM, and RDM. The minimum increase in the values of above mentioned parameters was 29.2, 56.3, and 63.6%, respectively by application of the highest conc. treatment Mp3+Co3 over the highest level of inoculum Mp3. While, maximum increase of 48.1, 65.3, and 75% in the values of aforesaid parameters, respectively was observed by application of the highest conc. of *Carthamus* powder in the treatment Mp1+Co3, over Mp1 (Fig 1 A-C).

3.3 Effects of treatments with *Carthamus oxyacantha* on pigments of maize

Photosynthetic pigments, such as Chlorophyll a, Chlorophyll b and carotenoids were decreased by the application of three levels of inoculum (Mp1, Mp2, and Mp3). This decrease was up to (31, 37, and 28 %), (38, 49, and 35 %), and (41, 55, and 39 %) in Chl a, Chl b, and carotenoids, respectively over C. However, the application of weed significantly increased the chlorophyll a, b and carotenoid contents. The values of Chl a, Chl b, and carotenoids were significantly increased in the treatments having weed powder amendments over the infected treatments (Mp1, Mp2 and Mp3). The minimum increase in the values of Chl a, Chl b, and carotenoids were seen in the treatment Mp3+Co3, which were 48, 58, and 34% for Chl a,

Chl b, and carotenoids, respectively, over Mp3. Whereas, the maximum increase in the values of Chl a, Chl b, and carotenoids were seen in the treatment Mp1+Co3, which were 58, 64, and 46% for Chl a, Chl b, and carotenoids, respectively, over the positive control treatment (Mp1) (Fig 2, A- C).

3.4 Effects of treatments with *Carthamus oxyacantha* on physiological attributes of maize

Carbon assimilation rate (*A*) and stomatal conductance (*gs*) were decreased up to (28, 34, and 47%) and (29, 33, and 45%), respectively for inoculated treatments (Mp1, Mp2, and Mp3) in comparison with non-inoculated treatment C. A significant decrease in (*A*) and (*gs*), were also observed for the treatments with autoclaved inoculums (Amp2 & Amp3). There was an increase of (2, 10, and 16%) for (*A*) with the treatments (Co1, Co2, and Co3) whereas, values of (*gs*) were significantly increased for Co2 and Co3 up to 17 and 25%, respectively, for treatments with weed powder (Co2, and Co3) in comparison to treatment C. There was maximum increase of 32, 45, and 54%, respectively for (*A*); 28, 44, and 58%, respectively for (*gs*), by treatments with weed powder (Mp1+Co1, Mp1+Co2, and Mp1+Co3), in comparison to inoculated treatment Mp1. There was minimum but significant increase of 22, 34, and 40%, respectively for (*A*); 22, 38, and 48%, respectively for (*gs*), by treatments with weed powder (Mp3+Co1, Mp3+Co2, and Mp3+Co3) in comparison with inoculated treatment Mp3 (Fig 3, A&B).

A significant decrease 20, 26, and 38% in rate of transpiration (*E*) was observed with the application of three different levels of inoculum such as Mp1, Mp2, and Mp3, respectively. Whereas, with the application of three different levels of *Carthamus* powder Co1, Co2, and Co3, the values of (*E*) progressively increased up to 5, 13, and 28% over C, respectively. Minimum increase of up to 3, 14, and 43% in infested treatments (Mp3+Co1, Mp3+Co2, and Mp3+Co3) was observed over inoculated treatment Mp3. Whereas, the maximum increase of up to 9, 25, and 48% in infested treatments (Mp1+Co1, Mp1+Co2, and Mp1+Co3) was observed over inoculated treatment Mp1 (Fig 4, A).

Internal carbon dioxide concentration (*Ci*) significantly increased up to 14, 25, and 32% with the application of Mp1, Mp2, and Mp3, respectively over C. Application of three different levels of *Carthamus* powder Co1, Co2, and Co3 also increased (*Ci*) up to 11, 17, and 21, respectively. Minimum significant increase of up to 7, 10, and 14% in infested treatments (Mp3+Co1, Mp3+Co2, and Mp3+Co3) was observed over inoculated treatment Mp3. Whereas, the maximum increase of up to 15, 19, and 20% in infested treatments (Mp1+Co1, Mp1+Co2, and Mp1+Co3) was observed over Mp1. There was significant decrease of 5 and 10%, for carbon dioxide concentration (*Ci*), for treatments (Amp2 & Amp3), respectively, over C (Fig 4, B).

3.5 Effects of treatments with *Carthamus oxyacantha* on defense related antioxidant enzymes of maize

Activities of antioxidant enzymes were noticeably enhanced in infected maize plants. Super oxide dismutase (SOD) activity was significantly increased up to 31.9, 55.3, and 79.4% due to pathogen infection for Mp1, Mp2, and Mp3, respectively over C. Application of *Carthamus* powder also increased the values of SOD significantly, up to 13.5, 25.2, and 39%, over C respectively, for Co1, Co2, and Co3. The minimum increase of 9.4% in the SOD activity were observed in the treatment Mp1+Co1, over Mp1. The maximum increase in the value of SOD was 26.7% for Mp3+Co3, over Mp3 (Fig 5, A).

Peroxidase (POD) activity was also significantly enhanced due to pathogen infection up to 9.1, 17.2, and 24.6% for Mp1, Mp2, and Mp3, respectively over C. The application of *Carthamus* powder also increased the values of POD significantly, up to 5.2, and 13.4% over C respectively, for Co2, and Co3. The minimum increase (17.4%) in the values of POD activities was seen in the treatment Mp1+Co1, over Mp1, while, the maximum increase was 28.4% for Mp3+Co3 over Mp3 (Fig 5, B).

Catalase (CAT) activities were also increased in infected treatments up to 47.2, 66.7, and 94.4% for Mp1, Mp2, and Mp3, respectively over C. Application of *Carthamus* powder also increased the values of CAT

significantly, up to 36.1, 46.3, and 63% over C, respectively, for Co1, Co2, and Co3. The minimum increase (25.2%) in CAT activity was seen in the treatment Mp1+Co1, over Mp1. On the contrary, the maximum increase was 28.1% for Mp3+Co3, over Mp3 (Fig 5, C)

4. Discussion

In the present study the soil amendment with an astraceous weed *Carthamus oxyacantha* was investigated against *Macrophomina phaseolina,* the cause of charcoal rot of maize variety (Neelam). The effect of different treatments was assessed in terms of morphological, physiological, and biochemical attributes of maize plants under pot conditions, in repeated set of trials, to investigate efficacy antifungal weed against charcoal rot of maize. Disease assessment, of charcoal rot infection in maize variety Neelam was based on the variables of disease incidence (DI), disease severity index (DSI), area under disease incidence progress curve (AUDIPC), and area under disease severity progress curve (AUDSPC). The occurrence of first symptom of charcoal rot was observed on infected plants, near tasseling stage of maize. Both disease incidence (DI) and disease severity index (DSI) were measured at three different times with 14 days of interval. Area under disease incidence progress curve (AUDIPC) and area under disease severity progress curve (AUDSPC) were observed on 70 DAS. There was no disease in negative control maize plant. When comparing three different strengths of inoculum, DI, DSI, AUDIPC, and AUDSPC recorded for Mp1, Mp2, and Mp3 were increased with increase of inoculum level of the pathogen in the soil. The remarkable infection level in infected plants may be linked to interruption of water and mineral upward flow in xylem vessels, owing to colonization of the stalk tissues by *M. phaseolina*. These results are in agreement with those reported by [36], who reported interruption of the conducting tissues by colonization of *Fusarium verticillioides,* which ultimately damage the flow of water and mineral salts in the conducting tissues. The similar interruption by infection of *Ceratocystis fimbriata* and *Ceratocystis smalleyi* in mango and bitternut hickory plants was reported by earlier workers [37, 38]. Previously, [39], tested different inoculum concentration of *Spilocaea oleaginea,* causing olive leaf spot (OLS). The disease severity of OLS, was

increased when inoculum concentration of *S. oleaginea* increased from 1.0×10^{2} to 2.5×10^{5}conidia mL^{-1}. It was also observed that percentages of DI and DSI were also increased with increasing days after sowing (DAS) in infected treatments. *C. oxyacantha* having antifungal potency against charcoal rot, suppressed DI and DSI up to 40 and 55%, over Mp3, respectively, in treatment (Mp3+Co3). AUDIPC and AUDSPC both were deceased with increasing concentrations of weed powder in selected treatments. In pot screening assays, antifungal activity of weed powder @ rate of 0.5, 1, and 1.5 % concentrations was assessed against the charcoal rot infection of maize caused by *M. phaseolina.* When different concentration of soil amendments of weed powder were used to control the disease infection, percentage of DI and DSI is decreased with increasing concentrations of weed powder, in selected treatments. Similar findings were reported by [40], where the application of decomposed neem leaves reduced the root rot incidence of cowpea, soil amendment with neem cake and farmyard manure (FYM) reduced the incidence of charcoal rot disease in chickpea and soybean [41].

Soil amendments with organic materials in crop fields is an efficient way to restore soil organic matter content and to improve soil quality directly or indirectly. Directly it is due to enhancing the availability of micro and macronutrients and indirectly by providing some biologically active compounds (antifungal compounds & antioxidants). Soil amendments with organic materials may alter the communities of microorganisms, which might be helpful to suppress harmful effects of other pathogens. Previous investigations indicate that organic amendments can reduce the incidence of diseases caused by soil borne pathogens including *M. phaseolina* [11, 42]. Soil amendments with different plant residues have different effect on the growth of other plants either by enhancing the growth of recipient plant or by retarding the growth of recipient plant. In present investigation, *C. oxyacantha* showed positive allelopathy for maize plants as well as antifungal efficacy to control charcoal rot in maize. Application of weed powder *C. oxyacantha* increased shoot length (SL), shoot dry mass (SDM) and root dry mass (RDM) over non-infested control (C). In previous investigation, [43] reported the positive allelopathy of *C. oxyacantha* for maize plants. Previous investigations have also similar findings, as application of dry leaf powder of *Acacia*

nilotica in pot of infested mash bean by *M. phaseolina,* @ rate of 1, 2 and 3% (W/W) increased SL, SFW, SDW, RFW & RDW up to 35, 96, 45, 92, and 74%, over non-treated infested control [44].

In general, autoclaved *M. phaseolina* also negatively affected the morphological, biochemical, and physiological attributes of maize and this effect was significant at the higher conc. It might be due to sorghum seeds used as substrate for inoculum multiplication and negative allelopathy of sorghum was reported in previous studies [45]. Similarly, [46] reported the negatively allelopathy of sorghum stem residue on maize plants.

The photosynthetic pigments such as chlorophyll a, chlorophyll b, and carotenoid were significantly reduced in infected plants over healthy plants (control) in pots as well as in field trials. Previous investigations, also reported fungal infection had inhibitory effects on synthesis of chlorophyll as well as carotenoids [47]. [12] reported similar trend of reduction in photosynthetic pigments by infection of *M. phaseolina* in mung bean. This reduction in synthesis of photosynthetic pigments is due to maximum utilization of energies to combat against fungal infection rather in the synthesis of pigments by infected plants. Soil amendments with weed powder of *C. oxyacantha*, enhanced the content of photosynthetic pigments. Previously, increase in photosynthetic pigments by addition of organic matter in soil was reported [14, 48].

Physiochemical alterations may also occur in plants by pathogens causing root infection and disturb the vascular system which ultimately effect plant growth by hindering the rate of photosynthesis and carbon assimilation by the plants [36, 38]. For many host-pathogen interactions, a decrease in rate of photosynthesis *(A)* was linked with lower stomatal conductance (*gs*) and higher internal CO_2 concentration (*Ci*) [36, 49]. The photosynthetic restrictions can be attributed to limitations on CO_2 fixation at the biochemical level and not only to reductions on CO_2 influx due to stomatal closure [36, 50]. Interestingly, in the present study, there was a progressive decline in (*A*), (*gs*), and (*E*) values while the (*Ci*) values increased in maize leaves in response to infection. Stomatal limitations may have contributed to the lower

(*A*) values since it was accompanied by decreases in (*gs*) values indicating, therefore, an imperceptible influx of CO_2 into the leaf tissues that could impair photosynthesis in leaves as a result of *M. phaseolina* infection on the stalk tissues. In the present study, maize plants infected with *M. phaseolina* resulted in great dehydration promoted by fungal colonization of the xylem vessels affecting (*gs*), (*Ci*), and (*E*) consequently lowering (*A*) due to stomatal closure [36, 51]. An interesting aspect *M. phaseolina* infection was an increase on (*Ci*) values indicating a behavior associated with lower activity of photosynthetic enzymes (e.g., RuBisCo) limiting CO_2 fixation at the chloroplasts level as reported by [52] for the infection of *Pyricularia orizae* in wheat plants. Moreover, reductions on (*A*) due to *M. phaseolina* infection are mainly associated with stomatal limitations coupled to loss of biochemical performance in the photosynthetic process. Application of weed powder (*C. oxyacantha*) having positive allelopathy for maize increased all physiological parameters over C. Similar findings were given by [53], who reported increase of 21, 22, and 21% in (*A*), (*gs*), and (*E*), over control, respectively, by the application of soybean cake. Similarly, [54] reported 62% increase in (*Ci*), over control by the soil amendment with moringa leaves in maize field. In our investigation, soil amendment with powder of *C. arvense* and *P. hysterophorus* decreased values of (*A*), (*gs*), (*E*), and (*Ci*).

Soil amendments with weed powders of *C. oxyacantha*, enhanced the contents of SOD, POD, and CAT activities. The harmful effects of reactive oxygen species (ROS) are minimized by increased activities of antioxidant enzymes such as superoxide dismutase (SOD), peroxidase (POD) and catalase (CAT). In fact, SOD, POD and CAT activities were increased in the maize plant tissues by *M. phaseolina*. In the present study, the addition of pathogen inoculum as well as amendment with weed resulted in higher SOD, CAT and POD activities. The higher antioxidant activities are considered beneficial for the plant as these enzyme activities help to remove the reactive oxygen species (ROS) generated in infected plant tissues or by soil amendments with plant residues. Similarly, previous investigations reported, SOD and POD activities were increased in maize plants infected by *Fusarium oxysporum* [36]. These results are in agreement with those reported by [50], who reported 79 to 176% increase in SOD activities over control by *M. phaseolina*

infection in wheat plants. Similar trend of increase in POD activities was also reported by [11], in which charcoal infection increased POD values up to 15%, over non-infested control. Present results of increase in CAT activities by pathogen infection have agreement with previous studies [11, 55].

5. Conclusion

In present investigation, *Carthamus oxyacantha* showed strong antifungal activity against *M. phaseolina*, causing charcoal rot in maize. Antifungal efficacy of *Carthamus* powder was increased by increasing conc. of *Carthamus* powder. Addition of *Carthamus* powder not only suppressed the charcoal rot in maize, it also enhanced SL, SDM and RDM of maize plants. Photosynthetic pigments were also increased in infested and non-infested plants by addition of *Carthamus* powder. Moreover, physiological parameters, (*A*), (*gs*), (*E*), and (*Ci*) were also enhanced in infested plants by soil amendment with *Carthamus* powder. Activities of SOD, POD and CAT were also increased in infested and non-infested plants by addition of *Carthamus* powder. Disease suppressing ability of *Carthamus* powder suggesting, *C. oxyacantha* might have active antifungal metabolites against *M. phaseolina*. Additionally, positive allelopathy of *C. oxyacantha* for maize, it might have nutrients and growth stimulating hormones which enhanced growth parameters in non-infested maize plants.

6. Recommendations

As soil amended with *C. oxyacantha* effectively controlled charcoal rot of maize in pot experiment and also showed compatibility with maize plants, therefore, *C. oxyacantha* can be utilized by farmers to control charcoal rot and increase per hectare yield in their fields.

Reference

1. Erenstein O, Jaleta M, Sonder K, Mottaleb K, Prasanna BM. Global maize production, consumption and trade: Trends and R&D implications. Food Security. 2022 Oct;14(5):1295-319.

2. Pakistan Agriculture research council; *Maize, Sorghum* and *Millet program.* [October 2022 Oct 12] Available from http://www.parc.gov.pk/

3. United States Department of Agriculture. *World Corn Production 2022/2023*. [October 2022 Oct 12] Available from, https://www.usda.gov/oce/ag-outlook-forum/2022-aof-program

4. Ngoune Tandzi L, Mutengwa CS. Estimation of maize (*Zea mays* L.) yield per harvest area: Appropriate methods. Agronomy. 2019 Dec 23;10(1):29.

5. Moore D, Robson GD, Trinci AP. 21st century guidebook to fungi. Cambridge University Press; 2020 May 8.

6. Kaur S, Dhillon GS, Brar SK, Vallad GE, Chand R, Chauhan VB. Emerging phytopathogen *Macrophomina phaseolina*: biology, economic importance and current diagnostic trends. Critical Reviews in Microbiology. 2012 May 1;38(2):136-51.

7. Das IK, Indira S, Annapurna A, Seetharama N. Biocontrol of charcoal rot in sorghum by fluorescent pseudomonads associated with the rhizosphere. Crop Protection. 2008 Nov 1;27(11):1407-14.

8. Hegde R, Desai S. A preliminary survey of incidence of stalk-rot complex of maize in two districts of Karnataka.

9. Pun KB, Doraiswamy S, Valluvaparidasan V. Studies on seed-borne nature of *Macrophomina phaseolina* in okra. Plant Disease Research. 1998;13:162-4.

10. Chaudhry QU. Climate change profile of Pakistan. Asian development bank; 2017 Aug 1.

11. Shoaib A, Munir M, Javaid A, Awan ZA, Rafiq M. Anti-mycotic potential of *Trichoderma* spp. and leaf biomass of *Azadirachta indica* against the charcoal rot pathogen, *Macrophomina phaseolina* (Tassi) Goid in cowpea. Egyptian Journal of Biological Pest Control. 2018 Dec;28:1-7.

12. Shoaib A, Khan KA, Awan ZA, Jan BL, Kaushik P. Integrated management of charcoal rot disease in susceptible genotypes of mungbean with soil application of micronutrient zinc and green manure (prickly sesban). Frontiers in Microbiology. 2022 Jul 25;13:899224.

13. Mawar R, Lodha S. Relative efficacy of on-farm weeds as soil-amendment for managing dry root rot of clusterbean in an arid environment. Phytopathologia Mediterranea. 2006 Dec 1;45(3):215-24.

14. Omara AE, Hafez EM, Osman HS, Rashwan E, El-Said MA, Alharbi K, Abd El-Moneim D, Gowayed SM. Collaborative impact of compost and beneficial rhizobacteria on soil properties,

physiological attributes, and productivity of wheat subjected to deficit irrigation in salt affected soil. Plants. 2022 Mar 25;11(7):877.

15. Aftab A, Yousaf Y, Javaid A, Riaz N, Younas A, Rashid M, Shamsher B, Arif A. Antifungal activity of vegetative methanolic extracts of *Nigella sativa* against *Fusarium oxysporum* and *Macrophomina phaseolina* and its phytochemical profiling by GC-MS analysis. Int. J. Agric. Biol. 2019 Jan 1;21(3):569-76.
16. Lorenzetti E, Heling AL, Carvalho J, Faria VD, Fujimoto JY, Stangarlin JR, Kuhn OJ. Antimicrobial activity of plant extracts on the development of *Macrophomina phaseolina* and influence of sterilization methods. Scientia Agraria Paranaensis. 2018;17(1):112-8.

17. Shi X, Cordero T, Garrigues S, Marcos JF, Daròs JA, Coca M. Efficient production of antifungal proteins in plants using a new transient expression vector derived from tobacco mosaic virus. Plant biotechnology journal. 2019 Jun;17(6):1069-80.

18. Singh, A., Patel, P., & Agrawal, P. K. Management of Collar Rot Disease Caused by *Macrophomina phaseolina* in Jatropha curcas-Green Diesel Plant. Journal of Plant Pathology & Microbiology. 2018;*9*(1):1-4.

19. Abid M, Hussain F. Screening of ethnomedicinal plants for their antifungal and nematicidal activities against soil-borne phytopathogens. South African Journal of Botany. 2022 Jul 1;147:18-23.

20. Wenzel S, Stolte H, Soose M. Effects of silibinin and antioxidants on high glucose-induced alterations of fibronectin turnover in human mesangial cell cultures. Journal of Pharmacology and Experimental Therapeutics. 1996 Dec 1;279(3):1520-6.

21. Zia, U. H. K., Shafiullah, K., Yongmei, C., & Pingyu, W. (2013). In vitro antimicrobial activity of the chemical constituents of *Cirsium arvense* (L). Scop. Journal of Medicinal Plants Research. 2013;*7*(25):1894-1898.

22. Rafiq M, Javaid A, Shoaib A. Possible antifungal and antibacterial constituents in inflorescence extract of Carthamus oxycantha. Mycopath. 2017;15(2):89-95.

23. Marian M, Voşgan Z, Roşca OM, Mihalescu L. Allelopathy relationship between plants and their use in organic farming. InIOP Conference Series: Materials Science and Engineering 2017 May 1 (Vol. 200, No. 1, p. 012039). IOP Publishing.

24. Mengistu A, Ray JD, Smith JR, Paris RL. Charcoal rot disease assessment of soybean genotypes using a colony-forming unit index. Crop Science. 2007 Nov;47(6):2453-61.

25. Pandey AK, Yee M, Win MM, Lwin HM, Adapala G, Rathore A, Sheu ZM, Nair RM. Identification of new sources of resistance to dry root rot caused by *Macrophomina phaseolina* isolates from India and Myanmar in a mungbean mini-core collection. Crop Protection. 2021 May 1;143:105569.

26. Pal KK, Tilak KV, Saxcna AK, Dey R, Singh CS. Suppression of maize root diseases caused by *Macrophomina phaseolina*, *Fusarium moniliforme* and *Fusarium graminearum* by plant growth promoting rhizobacteria. Microbiological research. 2001 Jan 1;156(3):209-23.

27. Iqbal U, Mukhtar T, Iqbal SM. *In vitro* and *in vivo* evaluation of antifungal activities of some antagonistic plants against charcoal rot causing fungus, *Macrophomina phaseolina*. Pakistan Journal of Agricultural Sciences. 2014 Sep 1;51(3):689-94.

28. Hanway, J.J. Growth stages of corn (*Zea mays* L.). Agronomy Journal, 1963;55:487-492

29. Bag, T. K. Use of metal salts in the control of *Sclerotium* rot of selected leguminous crop. MSc. Thesis, BCKVV. 1991; India.

30. Kone N, Asare-Bediako E, Silue S, Kone D, Koita O, Menzel W, Winter S. Influence of planting date on incidence and severity of viral disease on cucurbits under field condition. Annals of Agricultural Sciences. 2017 Jun 1;62(1):99-104.

31. Alharby HF, Al-Zahrani HS, Hakeem KR, Alsamadany H, Desoky ES, Rady MM. Silymarin-enriched biostimulant foliar application minimizes the toxicity of cadmium in maize by suppressing oxidative stress and elevating antioxidant gene expression. Biomolecules. 2021 Mar 21;11(3):465.

32. Pal G, Kumar K, Verma A, Verma SK. Seed inhabiting bacterial endophytes of maize promote seedling establishment and provide protection against fungal disease. Microbiological Research. 2022 Feb 1;255:126926.

33. El-Shafey NM, AbdElgawad HR. Antioxidants Released from *Cichorium pumilum* Jacq. Amendment Mitigate Salinity Stress in Maize. Jordan Journal of Biological Sciences. 2020 Oct 1;13(4).

34. Chen T, Zhang B. Measurements of proline and malondialdehyde content and antioxidant enzyme activities in leaves of drought stressed cotton. Bio-protocol. 2016 Sep 5;6(17):e1913.

35. Gao JF. Experimental guidance for plant physiology. China Higher Education Press: Beijing, China. 2006.

36. Cacique IS, Pinto LF, Aucique-Pérez CE, Wordell Filho JA, Rodrigues FA. Physiological and biochemical insights into the basal level of resistance of two maize hybrids in response to *Fusarium verticillioides* infection. Plant physiology and biochemistry. 2020 Jul 1;152:194-210.

37. Park JH, Juzwik J, Cavender-Bares J. Multiple *Ceratocystis smalleyi* infections associated with reduced stem water transport in bitternut hickory. Phytopathology. 2013 Jun;103(6):565-74.

38. Bispo WM, Araújo L, Bermúdez-Cardona MB, Cacique IS, DaMatta FM, Rodrigues FA. *Ceratocystis fimbriata* induced changes in the antioxidative system of mango cultivars. Plant Pathology. 2015 Jun;64(3):627-37.

39. Obanor FO, Walter M, Jones EE, Jaspers MV. Effects of temperature, inoculum concentration, leaf age, and continuous and interrupted wetness on infection of olive plants by *Spilocaea oleagina*. Plant Pathology. 2011 Apr;60(2):190-9.

40. Vengadeshkumar L, Kalaiselvi M, Meera T, Sanjayghandi S, Udhayakumar R, Rajamohan K, Sudhasha S. Effect of neem compared with *Pseudomonas fluorescens* on the management of cowpea root rot disease. Journal of Biopesticides. 2019 May 1;12(2):232-8.

41. Salman A, Backhouse D, Yunusa I. Using Neem to Control Charcoal Rot Of Chickpea. 2018.

42. Kumar KS, Balabaskar P, Sivakumar T, Kannan R, Saravanan KR. Bio–efficacy of culture filtrate of bacillus cereus against on the growth of *Macrophomina phaseolina* causing root rot of groundnut and different organic amendments on the survivability of *Bacillus cereus*. Plant Archives. 2020;20(1):1547-50.

43. Amir H., Waqas, A., & Muhammad, I. Evaluation of allelopathic effect of *Carthamus Oxyacantha* against wheat and maize seed germination. Letters in applied NanoBioScience. 2020;*9* :814-818.

44. Javaid A, Kanwal A, Shoaib A. Effect of *Trichoderma harzianum* and dry leaves of *Acacia nilotica* subsp. indica on growth of mash bean in *Macrophomina phaseolina* contaminated soil. Int. J. Biol. Biotechnol. 2018;15:535-40.

45. Wilson RG. Effect of Canada thistle (*Cirsium arvense*) residue on growth of some crops. Weed science. 1981 Mar;29(2):159-64.

46. Kim SY, De Datta SK, Robles RP, Kim KU, Lee SC, Shin DH. Allelopathic effect of sorghum extract and residues on selected crops and weeds. Korean Journal of Weed Science. 1994;14(1):34-41.

47. Shehbaz M, Rauf S, Al-Sadi AM, Nazir S, Bano S, Shahzad M, Hussain MM. Introgression and inheritance of charcoal rot (*Macrophomina phaseolina*) resistance from silver sunflower (*Helianthus argophyllus* Torr. & A. Gray) into cultivated sunflower *(Helianthus annuus* L.). Australasian Plant Pathology. 2018 Jul;47:413-20.

48. Shahkolaie SS, Baranimotlagh M, Dordipour E, Khormali F. Effects of inorganic and organic amendments on physiological parameters and antioxidant enzymes activities in *Zea mays* L. from a cadmium-contaminated calcareous soil. South African Journal of Botany. 2020 Jan 1;128:132-40.

49. Tatagiba SD, DaMatta FM, Rodrigues FÁ. Leaf gas exchange and chlorophyll a fluorescence imaging of rice leaves infected with *Monographella albescens*. Phytopathology. 2015 Feb;105(2):180-8.

50. Debona D, Rodrigues FÁ, Rios JA, Nascimento KJ. Biochemical changes in the leaves of wheat plants infected by Pyricularia oryzae. Phytopathology. 2012 Dec;102(12):1121-9.

51. Wang M, Sun Y, Sun G, Liu X, Zhai L, Shen Q, Guo S. Water balance altered in cucumber plants infected with *Fusarium oxysporum* f. sp. cucumerinum. Scientific reports. 2015 Jan 12;5(1):7722.

52. Rios JA, Aucique-Pérez CE, Debona D, Cruz Neto LB, Rios VS, Rodrigues FA. Changes in leaf gas exchange, chlorophyll a fluorescence and antioxidant metabolism within wheat leaves infected by *Bipolaris sorokiniana*. Annals of Applied Biology. 2017 Mar;170(2):189-203.

53. Ye S, Liu T, Niu Y. Effects of organic fertilizer on water use, photosynthetic characteristics, and fruit quality of pear jujube in northern Shaanxi. Open Chemistry. 2020 Jun 2;18(1):537-45.

54. Afzal I, Imran S, Javed T, Basra SM. Evaluating the integrative response of moringa leaf extract with synthetic growth promoting substances in maize under early spring conditions. South African Journal of Botany. 2020 Aug 1;132:378-87.

55. Zhang M, Zhang C, Zhang S, Yu H, Pan H, Zhang H. *Klebsiella jilinsis* 2N3 promotes maize growth and induces resistance to northern corn leaf blight. Biological Control. 2021 May 1;156:104554.

Figure Click here to access/download;Figure;figures.docx

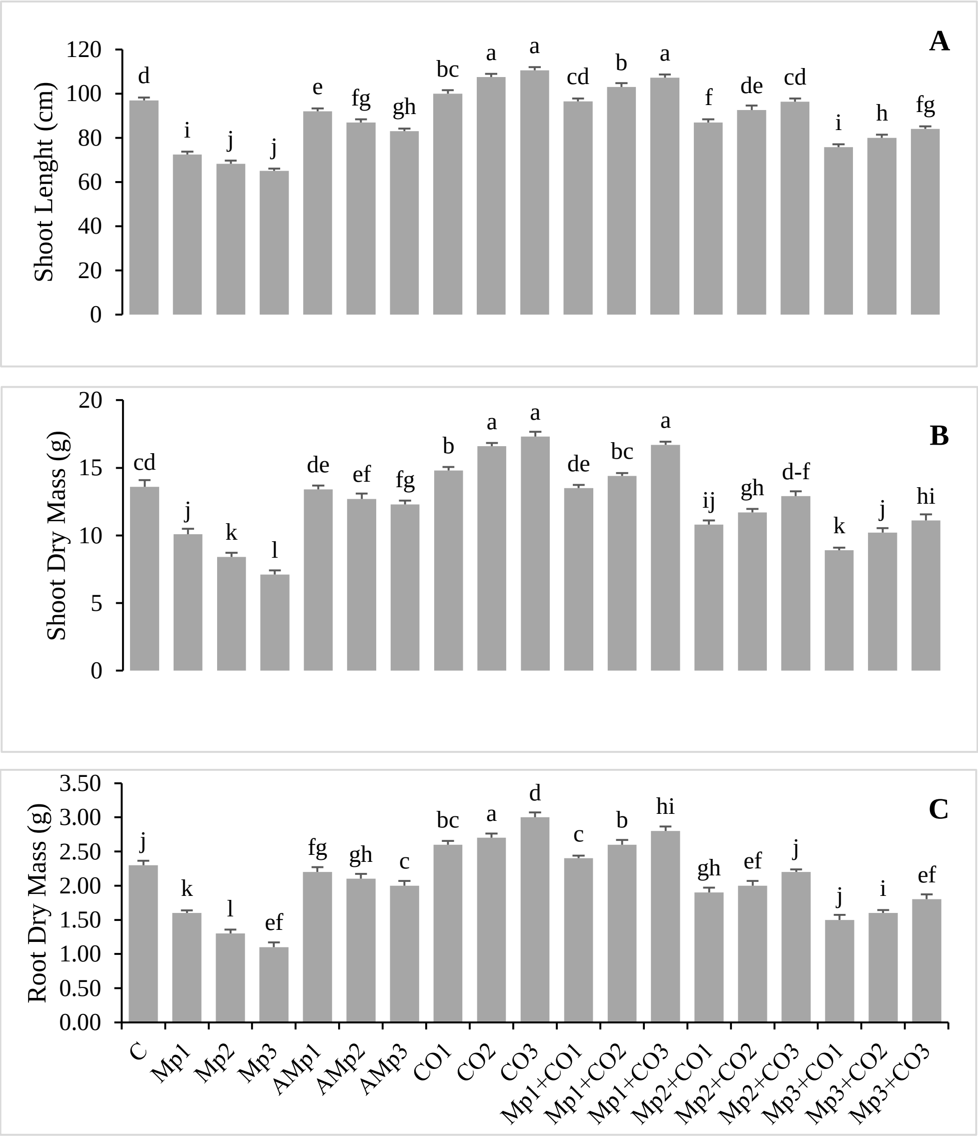

Fig-1: Effect of treatments on **(A)** shoot length, **(B)** shoot dry mass, and **(C)** root dry mass of maize in pot trials. Data represent means ± standard error of 5 replicates. Error bars with a common alphabet do not differ significantly at *P*=5% as computed by Fisher's LSD test, using Minitab 20.2.

Abbreviations: **C**: control (Without pathogen and soil amendment), **Mp**: *Macrophomina phaseolina*, **AMp**: Autoclaved *M. phaseolina*, **Co**: *Carthamus oxyacantha*, **Mp1**: Mp (1.2×10^5), **Mp2**: Mp (2.4×10^5), **Mp3**: Mp (3.6×10^5), **AMp1**: AMp (1.2×10^5), **AMp2**: AMp (2.4×10^5), **AMp3**: AMp (3.6×10^5), **Co1**: Co0.5%, **Co2**: Co1%, **Co3**: Co1.5%, **Mp1+Co1**: Mp (1.2×10^5)+Co0.5%, **Mp1+Co2**: Mp (1.2×10^5)+Co1%, **Mp1+Co3**: Mp (1.2×10^5)+Co1.5%, **Mp2+Co1**: Mp (2.4×10^5)+Co0.5%, **Mp2+Co2**: Mp (2.4×10^5)+Co1%, **Mp2+Co3**: Mp (2.4×10^5)+Co1.5%, **Mp3+Co1**: Mp (3.6×10^5)+Co0.5%, **Mp3+Co2**: Mp (3.6×10^5)+Co1%, **Mp3+Co3**: Mp (3.6×10^5)+ Co1.5%.

Note: Mp concentrations are given in colony forming units (CFU mL^{-1}).

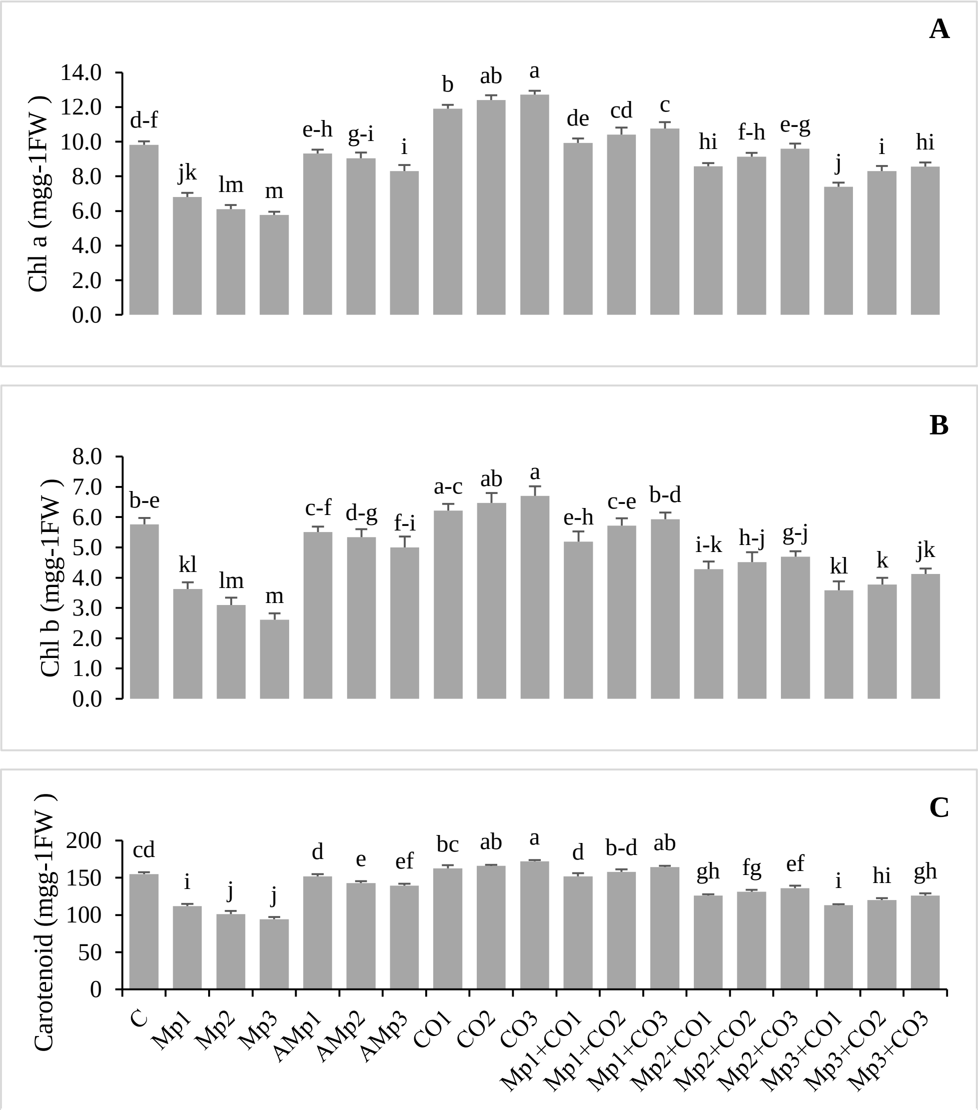

Fig 2: Effect of treatments on **(A)** chlorophyll a, **(B)** chlorophyll b, and **(C)** carotenoids of maize in pot trials. Data represent means ± standard error of 5 replicates. Error bars with a common alphabet do not differ significantly at *P*=5% as computed by Fisher's LSD test, using Minitab 20.2.

Abbreviations: **C**: control (Without pathogen and soil amendment), **Mp**: *Macrophomina phaseolina*, **AMp**: Autoclaved *M. phaseolina*, **Co**: *Carthamus oxyacantha*, **Mp1**: Mp (1.2×10^5), **Mp2**: Mp (2.4×10^5), **Mp3**: Mp (3.6×10^5), **AMp1**: AMp (1.2×10^5), **AMp2**: AMp (2.4×10^5), **AMp3**: AMp (3.6×10^5), **Co1**: Co0.5%, **Co2**: Co1%, **Co3**: Co1.5%, **Mp1+Co1**: Mp (1.2×10^5)+Co0.5%, **Mp1+Co2**: Mp (1.2×10^5)+Co1%, **Mp1+Co3**: Mp (1.2×10^5)+Co1.5%, **Mp2+Co1**: Mp (2.4×10^5)+Co0.5%, **Mp2+Co2**: Mp (2.4×10^5)+Co1%, **Mp2+Co3**: Mp (2.4×10^5)+Co1.5%, **Mp3+Co1**: Mp (3.6×10^5)+Co0.5%, **Mp3+Co2**: Mp (3.6×10^5)+Co1%, **Mp3+Co3**: Mp (3.6×10^5)+ Co1.5%.

Note: Mp concentrations are given in colony forming units (CFU mL^{-1}).

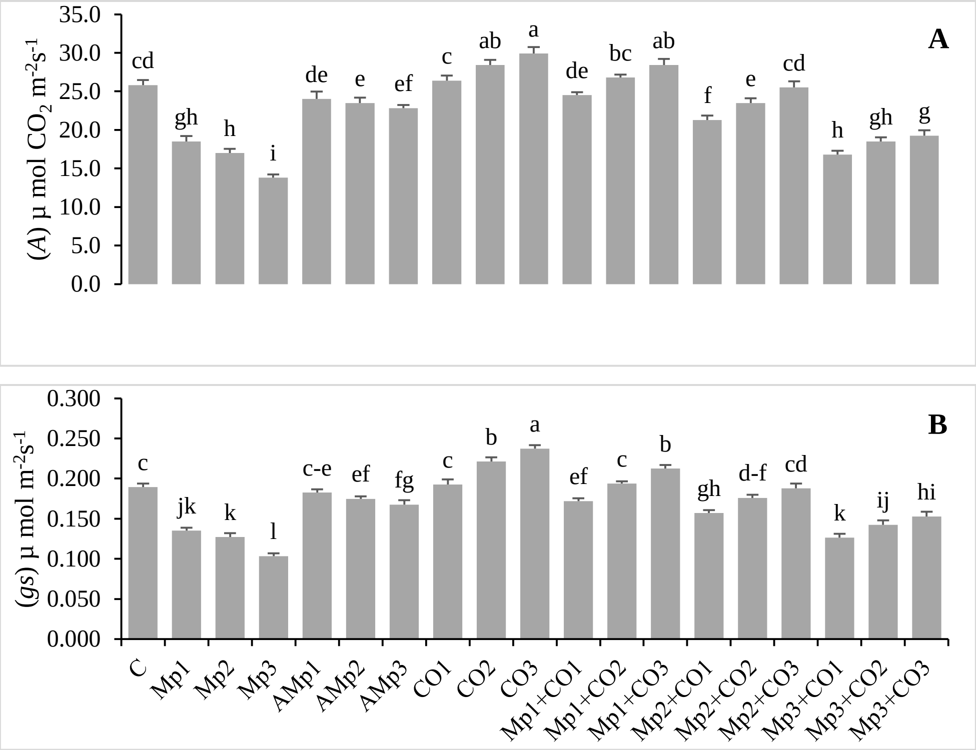

Fig 3: Effect of treatments on **(A)** rate of carbon assimilation, and **(B)** stomatal conductance of maize in pot trials. Data represent means ± standard error of 5 replicates. Error bars with a common alphabet do not differ significantly at *P*=5% as computed by Fisher's LSD test, using Minitab 20.2.

Abbreviations: **C**: control (Without pathogen and soil amendment), **Mp**: *Macrophomina phaseolina*, **AMp**: Autoclaved *M. phaseolina*, **Co**: *Carthamus oxyacantha*, **Mp1**: Mp (1.2×10^5), **Mp2**: Mp (2.4×10^5), **Mp3**: Mp (3.6×10^5), **AMp1**: AMp (1.2×10^5), **AMp2**: AMp (2.4×10^5), **AMp3**: AMp (3.6×10^5), **Co1**: Co0.5%, **Co2**: Co1%, **Co3**: Co1.5%, **Mp1+Co1**: Mp (1.2×10^5)+Co0.5%, **Mp1+Co2**: Mp (1.2×10^5)+Co1%, **Mp1+Co3**: Mp (1.2×10^5)+Co1.5%, **Mp2+Co1**: Mp (2.4×10^5)+Co0.5%, **Mp2+Co2**: Mp (2.4×10^5)+Co1%, **Mp2+Co3**: Mp (2.4×10^5)+Co1.5%, **Mp3+Co1**: Mp (3.6×10^5)+Co0.5%, **Mp3+Co2**: Mp (3.6×10^5)+Co1%, **Mp3+Co3**: Mp (3.6×10^5)+ Co1.5%.

Note: Mp concentrations are given in colony forming units (CFU mL^{-1}).

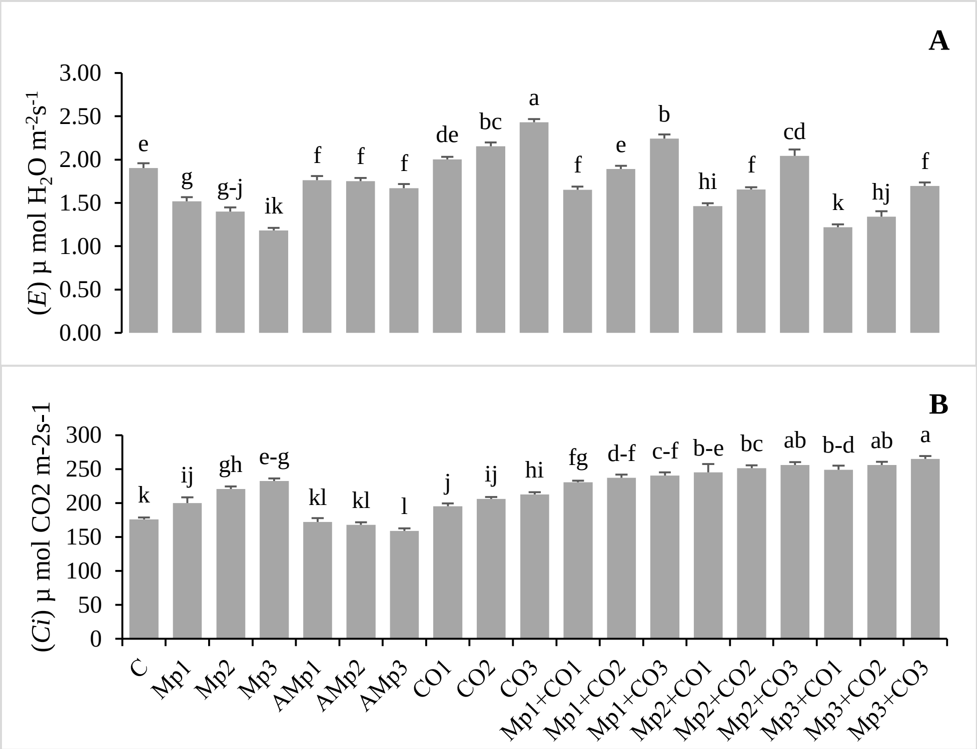

Fig 4: Effect of treatments on **(A)** transpiration rate, and **(B)** internal carbon dioxide conc. of maize in pot trials. Data represent means ± standard error of 5 replicates. Error bars with a common alphabet do not differ significantly at *P*=5% as computed by Fisher's LSD test, using Minitab 20.2.

Abbreviations: **C**: control (Without pathogen and soil amendment), **Mp**: *Macrophomina phaseolina*, **AMp**: Autoclaved *M. phaseolina*, **Co**: *Carthamus oxyacantha*, **Mp1**: Mp ($1.2×10^5$), **Mp2**: Mp ($2.4×10^5$), **Mp3**: Mp ($3.6×10^5$), **AMp1**: AMp ($1.2×10^5$), **AMp2**: AMp ($2.4×10^5$), **AMp3**: AMp ($3.6×10^5$), **Co1**: Co0.5%, **Co2**: Co1%, **Co3**: Co1.5%, **Mp1+Co1**: Mp ($1.2×10^5$)+Co0.5%, **Mp1+Co2**: Mp ($1.2×10^5$)+Co1%, **Mp1+Co3**: Mp ($1.2×10^5$)+Co1.5%, **Mp2+Co1**: Mp ($2.4×10^5$)+Co0.5%, **Mp2+Co2**: Mp ($2.4×10^5$)+Co1%, **Mp2+Co3**: Mp ($2.4×10^5$)+Co1.5%, **Mp3+Co1**: Mp ($3.6×10^5$)+Co0.5%, **Mp3+Co2**: Mp ($3.6×10^5$)+Co1%, **Mp3+Co3**: Mp ($3.6×10^5$)+ Co1.5%.

Note: Mp concentrations are given in colony forming units (CFU mL^{-1}).

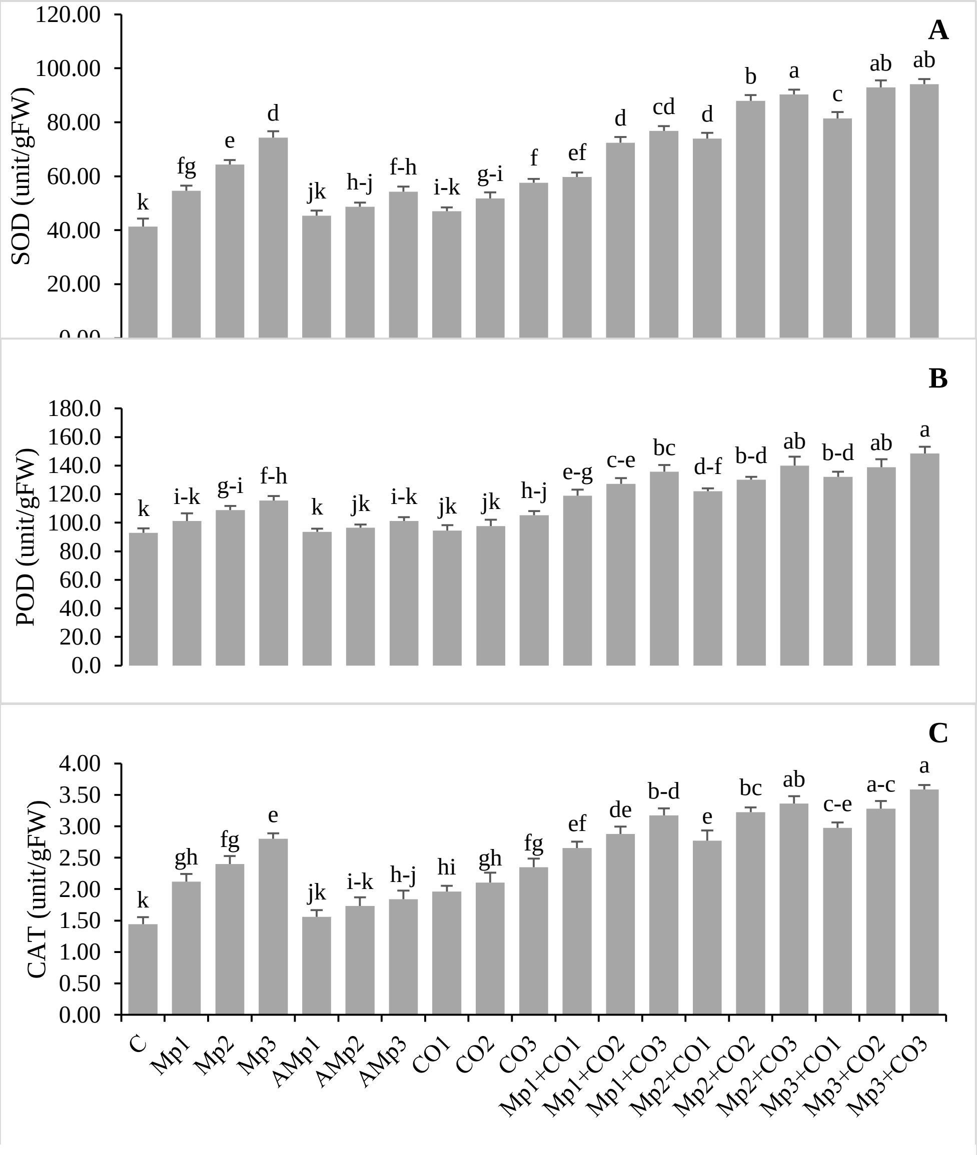

Fig 5: Effect of treatments on **(A)** superoxide dismutase, and **(B)** peroxidase, and **(C)** catalase activities of maize in pot trials. Data represent means ± standard error of 5 replicates. Error bars with a common alphabet do not differ significantly at *P*=5% as computed by Fisher's LSD test, using Minitab 20.2.

Abbreviations: **C**: control (Without pathogen and soil amendment), **Mp**: *Macrophomina phaseolina*, **AMp**: Autoclaved *M. phaseolina*, **Co**: *Carthamus oxyacantha*, **Mp1**: Mp (1.2×10^5), **Mp2**: Mp (2.4×10^5), **Mp3**: Mp (3.6×10^5), **AMp1**: AMp (1.2×10^5), **AMp2**: AMp (2.4×10^5), **AMp3**: AMp (3.6×10^5), **Co1**: Co0.5%, **Co2**: Co1%, **Co3**: Co1.5%, **Mp1+Co1**: Mp (1.2×10^5)+Co0.5%, **Mp1+Co2**: Mp (1.2×10^5)+Co1%, **Mp1+Co3**: Mp (1.2×10^5)+Co1.5%, **Mp2+Co1**: Mp (2.4×10^5)+Co0.5%, **Mp2+Co2**: Mp (2.4×10^5)+Co1%, **Mp2+Co3**: Mp (2.4×10^5)+Co1.5%, **Mp3+Co1**: Mp (3.6×10^5)+Co0.5%, **Mp3+Co2**: Mp (3.6×10^5)+Co1%, **Mp3+Co3**: Mp (3.6×10^5)+ Co1.5%.

Note: Mp concentrations are given in colony forming units (CFU mL^{-1}).
