# Supplementary material for: Allelopathic interactions of Carthamus oxyacantha, Macrophomina phaseolina and maize: Implications for the use of Carthamus oxyacantha as a natural disease management strategy in maize
Source: PLoS One. 2024 Oct 31;19(10):e0307082. doi: 10.1371/journal.pone.0307082 (PMC11527155; doi:10.1371/journal.pone.0307082)
Supplement: Attachment — Submitted filename: Response to Reviewers.docx [file pone.0307082.s010.docx]

**Response to the Comments**

**PONE-D-23-34629**

Respected Editor (Dr. Abhay K. Pandey) & Reviewers,

The authors are thankful to honorable editor and reviewers for sparing their precious time to review/improve our manuscript **“*Carthamus oxyacantha* as bio-elicitor and natural defense in maize against charcoal rot disease caused by *Macrophomina phaseolina*”**. Keeping in mind the comments of the honorable reviewer 2 (Dr. ALBERT MAIBAM), the authors revised the title to best depict the research work done & presented in this MS as **“Allelopathic interactions of *Carthamus oxyacantha, Macrophomina phaseolina* and maize; Implications for the use of *Carthamus oxyacantha* as a natural disease management strategy in maize”.**

The authors have revised the manuscript in track change mode.

**Request:** Please note that the authors have included one more author Anna Andolfi, cosupervisor of PhD thesis of Nazir Aslam. Previously there was a confusion about to include only the name of scholar and the main supervisor in the MS required for the award of PhD degree, now the confusion has been resolved as we need to include the name of cosupervisor also. We realize that PLOS One policy is very strict and there are numerous manuscripts of many authors retracted in the past, so the authors request to accept the inclusion of 3^rd^ author and hope this will not affect the integrity of review process/authorship. Thanks

Reply to the comments are presented below;

**Reviewer 1**

**Abstract and text:**

**Comment 1.**

Line 13: ‘A total of 19 treatments’

***Response:*** Now it is amended as pointed out.

**Comment 2.**

Line 16: Rephrase ‘dead inoculum’
***Response:*** ‘dead inoculum’ is replaced by autoclaved *M. phaseolina*. It was already discussed as autoclaved *M. phaseolina* in main body.

**Comment 3.**

Line 17 and onwards: Spell out the word ‘conc.’

***Response:*** The authors spelled out the word ‘conc.’ and rephrased it in all the onward text.

**Comment 4.**

SOD, POD, CAT and other acronyms: Do not abbreviate these in the abstract. Instead, write the complete words and their abbreviations the first time they are mentioned in the main text. Use the abbreviations onwards.

***Response:*** The authors revised and made changes to the manuscript and SOD, POD, CAT and other acronyms fully described at first mention.

**Introduction:**

**Comment 5.**

Good information about maize and M. phaseolina was provided. Adding some information about *C. oxyacantha* will be helpful, especially for readers that are not familiar with this weed.

***Response:*** The authors added information about *C. oxyacantha* in introduction portion of the manuscript.
**Comment 6.**

Lines 40 and 45: Not necessary to repeat that *M. phaseolina* is a seed and soil borne pathogen.

***Response:*** Removed the suggested repeated lines from the manuscript.

**Comment 7.**

Lines 49-50: Rephrase “climate change is very favorite for *M. phaseolina*”
***Response:*** Mentioned text have been rephrased.

**Comment 8**

Line 66: Spell out the word “1st”

***Response:*** Now the word “1^st^” is replaced by “first”.

**Materials and Methods:**

Some sections need additional details for readers to follow how the study was evaluated/conducted.
**Comment 9**

Lines 69-71: Add specific details/criteria on how *Carthamus oxyacantha* was identified.

***Response:*** The weed *C. oxyacantha* was identified based on its vegetative and floral characters and this information is now added in the manuscript.
**Comment 11**

Lines 73-75: How was the pathogen identified as *Macrophomina phaseolina*?

***Response:*** The *Macrophomina phaseolina* was identified by recording its morphological characters such as hyphae shape, colony color, presence of microsclerotia and size of microsclerotia and these specifications were also added in the manuscript.
**Comment 12**

Lines 75-76: Provide a short summary of the fungal inoculum preparation.

***Response:*** A short summary inoculum preparation has been added in manuscript.
**Comment 13**

Lines 119-123: What does “G” in equation 2 represent?

***Response:*** G’ stands for number of grading. This information added in the MS
**Comment 14**

Lines 137-142: What equipment/machine was used to measure the pigments?

***Response:*** The optical density was recorded at 663, 645, and 440.5 nm wavelength, respectively by using a spectrophotometer (Model UV 3000) and it is also mentioned in the revised manuscript.

**Discussion:**

**Comment 15** Indicate author citation for those written as part of the sentence: [36], [39], [40], [43], [46], [12], etc.

***Response:*** Added author citations for those scientific names written as part of the sentence: [36], [39], [40], [43], [46], [12] and all others at their first mention in the manuscript.

**Comment 16**
Spell out “@”

***Response:*** Spelled out “@” as “at the rate of” in the manuscript.
**Comment 17**

Proofread the abstract and introduction to make statements clear and unambiguous.
***Response:*** Proof read and updated the manuscript according to the reviewer’s comments/suggestions.

**Comment 18**

Add line numbers for page 11 and onwards.

***Response:*** line numbers are added in the revised manuscript.

**Reviewer 2**

**Comment 1**

Manuscript is written decently but lack molecular analysis to support biochemical assay, there is no specific bioelicitor and known marker genes to address the complex network associated with the particular defense mechanism.

**Response:**

The authors are highly obliged for appreciation and encouragement and for providing new insights for related work. The authors modified the title to match the research done as the title was misleading as the reader might think of molecular analysis to support biochemical assays, specific bioelicitor and known marker genes to address the complex network associated with the particular defense mechanism.

**Comment 2**

Used methods unable to correlate or justify the title.

**Response:** The title has been modified to depict/justify the research work done.

**Comment 3**

Try to avoid abbreviation usage in Abstract. Please explain Mp

***Response:*** All abbreviations are now fully described at first mention (abbreviated thereafter) in the abstract as well as in the whole MS. Also pointed out by the reviewer 1.

**Comment 4**

Estimation of Morphological and biochemical properties generally used as a supplementary data to cross check molecular study. So, addition of molecular analysis (known marker gene) will make complete conclusion.

***Response:*** The confusion arose due to the inappropriate title. Now the title has been revised to represent the actual work done/presented in the MS. By modifying the title comment 2 was also addressed. The authors are highly obliged for useful comment of addition of molecular analysis (known marker gene). The author will consider including such investigations in future studies.

**Comment 5**

It better to have particular selected active antifungal metabolite

***Response:***

Antifungal activity of *C. oxyacantha* has been reported and also the active compounds have been reported as per references 21 & 22. Although, *in vitro* antifungal activity of *C. oxyacantha* has been reported, its effectiveness has never been tested against charcoal rot of maize in *in-vivo* as well as its compatibility for maize were missing also. Therefore, pot experiment was conducted to investigate compatibility, antifungal efficacy of *C. oxyacantha*, to control charcoal rot of maize caused by *M. phaseolina*. Moreover, effect of soil amendment with *C.* *oxyacantha*, on morphological, physiological, and defense related attributes of maize were also investigated 1st time in this study.

*Comments closed:*

Regards

**Dr. Muhammad Akbar**

Associate Professor,

Department of Botany, University of Gujrat, Gujrat, 50700, Punjab, Pakistan.

[muhammad.akbar@uog.edu.pk](mailto:muhammad.akbar@uog.edu.pk) Mobile: +923337645058
