# Supplementary material for: Allelopathic interactions of Carthamus oxyacantha, Macrophomina phaseolina and maize: Implications for the use of Carthamus oxyacantha as a natural disease management strategy in maize
Source: PLoS One. 2024 Oct 31;19(10):e0307082. doi: 10.1371/journal.pone.0307082 (PMC11527155; doi:10.1371/journal.pone.0307082)
Supplement: Attachment — Submitted filename: Response to reviewers.docx [file pone.0307082.s011.docx]

Dated: May 20, 2024

**Response to the Comments**

**PONE-D-23-34629R1]**

Respected Editor (Dr. Abhay K. Pandey) & Reviewers,

The authors are thankful to honorable editor and reviewers for sparing their precious time to review/improve our manuscript **““Allelopathic interactions of *Carthamus oxyacantha, Macrophomina phaseolina* and maize; Implications for the use of *Carthamus oxyacantha* as a natural disease management strategy in maize”**. The authors have revised the manuscript in track change mode.

Reviewer's Responses to Questions

**Comments to the Author**

1. If the authors have adequately addressed your comments raised in a previous round of review and you feel that this manuscript is now acceptable for publication, you may indicate that here to bypass the “Comments to the Author” section, enter your conflict of interest statement in the “Confidential to Editor” section, and submit your "Accept" recommendation.

Reviewer #1: All comments have been addressed

Reviewer #2: All comments have been addressed

2. Is the manuscript technically sound, and do the data support the conclusions?

The manuscript must describe a technically sound piece of scientific research with data that supports the conclusions. Experiments must have been conducted rigorously, with appropriate controls, replication, and sample sizes. The conclusions must be drawn appropriately based on the data presented.

Reviewer #1: Yes

Reviewer #2: No

**Reply:** The authors have tried their level best to improve the conclusions section to show more clearly that conclusions have been drawn appropriately based on the data presented.

3. Has the statistical analysis been performed appropriately and rigorously?

Reviewer #1: Yes

Reviewer #2: No

**Reply:** The authors have tried their level best to choose the appropriate data analysis suitable to show the effects of different treatments on numerous parameters like morphological, physiological, biochemical and disease. These statistical tests included ANOVA followed by Fisher’s LSD test at 5% probability using computer software Minitab 20. (This portion has already included in the MS. The Minitab analysis files are shared as (supporting data).

The authors also tried to construct the heat map shown below using R programming but the authors realize that this test will not be appropriate to show the effects of different treatments on various parameters and is not suitable for such type of data where multiple treatments with their respective controls are given.

The authors also performed PCA analysis (using OriginPro 2024) of the data that shows the effect of different treatments on various parameters better as compared to heat map, so the authors included the PCA analysis in the revised MS. However, the authors think that graphs with LSD presented better show the effects of different treatments on different parameters.

4. Have the authors made all data underlying the findings in their manuscript fully available?

The [PLOS Data policy](http://www.plosone.org/static/policies.action#sharing) requires authors to make all data underlying the findings described in their manuscript fully available without restriction, with rare exception (please refer to the Data Availability Statement in the manuscript PDF file). The data should be provided as part of the manuscript or its supporting information, or deposited to a public repository. For example, in addition to summary statistics, the data points behind means, medians and variance measures should be available. If there are restrictions on publicly sharing data—e.g. participant privacy or use of data from a third party—those must be specified.

Reviewer #1: Yes

Reviewer #2: Yes

5. Is the manuscript presented in an intelligible fashion and written in standard English?

PLOS ONE does not copyedit accepted manuscripts, so the language in submitted articles must be clear, correct, and unambiguous. Any typographical or grammatical errors should be corrected at revision, so please note any specific errors here.

Reviewer #1: Yes

Reviewer #2: Yes

6. Review Comments to the Author

Please use the space provided to explain your answers to the questions above. You may also include additional comments for the author, including concerns about dual publication, research ethics, or publication ethics. (Please upload your review as an attachment if it exceeds 20,000 characters)

Reviewer #1: The authors have addressed my previous comments and made changes to significantly enhance the clarity of the manuscript. Please see minor comments below:

Reply to the comments are presented below;

**Reviewer 1**

**Comment 1.**

**Title:** Use colon instead of semicolon.

***Response:*** Now it is amended as pointed out and put the colon to replace semicolon.

**Comment 2.**

**Line 28:** Change to ‘48.1, 65.3, and 75.0%’ (or 48, 65, and 75%) to make decimal values consistent. Do the same with all values throughout the manuscript.

***Response:*** Now decimal values are consistent throughout the manuscript as per your guidance.

**Comment 3.**

**Line 98:** spell out ‘PDA’
***Response:*** PDA changed with potato dextrose agar.

**Comment 4.**

**Line 117:** Change to: ‘is given in Table 1.’

***Response:* “**is given in the table 1” have been corrected to ‘is given in Table 1.’

**Comment 5.**

**Line 135:** ‘between 5 and 6’

***Response:* “**between 5 & 6” have been corrected with ‘between 5 and 6’.

**Comment 6.**
**Line 153-155:** ‘Morphological parameters were measured after harvesting the plants on 80 DAS. Shoot length, shoot dry weight, and root dry weight of all plants in all treatments were recorded after oven drying at 70 ºC until a constant dry weight reading was achieved [31].’

***Response:*** The paragraph “Morphological parameters shoot length was measured by measuring tap, shoot dry weight and root dry weight of all plants of all treatments were recorded after oven drying at 70 ºC, till constant dry weight reading, after harvesting on 80 DAS [31]” has been replaced with ‘Morphological parameters were measured after harvesting the plants on 80 DAS. Shoot length, shoot dry weight, and root dry weight of all plants in all treatments were recorded after oven drying at 70 ºC until a constant dry weight reading was achieved [31].’

**Comment 7.**

**Line 173:** ‘and the following’

***Response:*** “the” has been added and corrected the sentence as: ‘and the following’

**Comment 8.**

**Line 175:** ‘the measurements were made between 10:00 AM and 2:00 PM’
***Response:*** “from 10:00 am to 2:00 pm” has been changed with ‘between 10:00 AM and 2:00 PM’

**Comment 9.**

**Line 179:** ‘The leaf powder was homogenized by adding 3 mL of chilled…’

***Response:*** The sentence “Homogenized the leaf powder by adding 3 ml of chilled…’ has been corrected and replaced with “The leaf powder was homogenized by adding 3 mL of chilled…’

**Comment 10.**

**Line 180:** ‘two centrifuge tubes, the supernatant was centrifuged at…’
***Response:*** ‘two centrifuge tubes, centrifuged at 10,000 *x g* for 20 min at 4 °C” corrected as ‘two centrifuge tubes, the supernatant was centrifuged at…’

**Comment 11.**

**Line 184-185:** ‘Crude enzyme solution (50 uL) from each sample…

***Response:*** the “crude enzyme “has been deleted and now this sentence has been corrected as ‘Crude enzyme solution (50 uL) from each sample…

**Comment 12**

Line 187-189. Rephrase because the sentences read like laboratory protocol.

***Response:*** Mentioned sentences are now rephrased.

**Comment 13.**

**Line 196:** Rewrite the following phrase because it reads like a laboratory protocol = heat and stir well, add 36.1 uL of 30% H_2_0_2_ after cooling.

***Response:*** Mentioned phases are rephrased.

**Comment 14**

**Line 203-204:** ‘and 1 mL of the reaction solution into the cuvette’

***Response:*** “were mixed” has been deleted from the sentence and now corrected sentence is “‘and 1 mL of the reaction solution into the cuvette’

**Comment 15**
**Line 219:** ‘and Mp3 increased progressively with increasing inoculum levels from 60 up to 100% for DI’

***Response:*** The sentence “and Mp3 were increased progressively with stages from 60 up to 80% for DI “has been replaced with “and Mp3 increased progressively with increasing inoculum levels from 60 up to 80% for DI’. Not 100% as per our disease measurements.

**Comment 16**

**Results:** Include the Table or Figure number at the end of first sentence where the result of the Table or Figure is mentioned. This is in addition to the Table or Figure number you included at the end of each paragraph.

***Response:*** Table or Figure number at the end of first sentence included in the revised Manuscript.

**Comment 17.**

**Line 262:** ‘Amp2 and Amp3’

***Response:***. The sign “&“ between Amp2 and Amp3 has been replaced with word “and”.

**Comment 18**

**Line 270:** ‘A significant decrease of 20…’

***Response:*** The word “of “has been added before 20 and now updated in the revised manuscript.

**Comment 19**

**Line 279:** ‘and 21%, respectively’

***Response:*** “%” has been added in the sentence.

**Comment 20**

**Discussion:** No need to repeatedly write the complete names of previously abbreviated terms.

***Response:*** Abbreviations are now fully described at 1^st^ mention in the MS the used only the abbreviations except in table 1 where each species was given with full genus name at first mention, as table needs to be self-explanatory.

**Comment 21**
**Line 345:** Write author citation for [45]

***Response:*** Author citation for [45] mentioned in revised manuscript at its first mention in the abstract, then abbreviated onward as per comment 20 above.

**Reviewer #2:**

**Comment 1**

As you mention, you have referred reference 21 and 22. There they have mentioned clearly the extracted chemical that can further process in detail studies regarding the allelopathic interaction. Why you have used the crude extract as to compare the associated data. It shows us that the work undertaken is very preliminary, not so rigorous.

***Response:***

Reference [21] discusses about antifungal compounds isolated from *Cirsium arvense* (L). Scop. These authors discussed only the *in vitro* antifungal activity of compounds isolated from *C. arvense*. They did not report the *in vivo* disease suppressing efficacy of *C. arvense*. **(***Carthamus oxyacantha* was mistakenly mentioned there in the introduction as reference [21] was about *C.* *arvense,* while reference [22] was specific to *C. oxyacantha,* both belonging to family Asteraceae*.* The authors of reference 22 mentioned the antifungal compound γ-Sitosterol, so reference 22 was retained and old ref [21] was removed and updated latest paper specific to *C. oxyacantha*.

Only few reports are also available about the presence of antifungal compounds in *C. oxyacantha* and now we have replaced the reference with reference pertinent to *Carthamus oxyacantha.* The added reference also report the presence of antifungal compounds in *C. oxyacantha* just as references [21] in previous version of the MS).

We have not used the crude extract of *C. oxyacantha*, instead we used the plant biomass as soil amendment.

In these references researchers mentioned about presence of antifungal compound in *C. oxyacantha* family Asteraceae, but not antifungal efficacy against charcoal rot causal agent *Macrophomina phaseolina*, it is first *in vivo* study in which *C. oxyacantha* was investigated against charcoal rot of maize. Moreover, the isolated natural compounds can not be tested *in vivo* because of low quantities purified through chromatographic procedures and these can only be tested *in vitro* but our present study focused on various other aspects of *in vivo* studies.

In addition to evaluating the antifungal efficacy at 3 different concentrations, the authors also included treatments to observe the allelopathic effects of not only the autoclaved pathogen but also the weed, *C. oxyacantha,* not investigated before in detail. Moreover, the authors also confirmed the allelopathic effects in terms of physiochemical attributes, not reported earlier. Therefore, the present study can be viewed as advanced study as it not only extended the studies of earlier researchers but also presented an improved way to investigate the allelopathic interactions. This need was also pointed out in a study reference [23], in a broader sense.

Thanks for your consideration.

**Comments closed:**
